# Flash Communication: On the Bistabilities and Reactivities of *ortho*-Phenylene Compounds with Ch=O→B Interactions

**DOI:** 10.1021/acs.organomet.5c00229

**Published:** 2025-08-05

**Authors:** Brendan L. Murphy, François P. Gabbaï

**Affiliations:** Department of Chemistry, Texas A&M University, College Station, Texas 77843-3255, United States

## Abstract

Intramolecular Lewis adducts, especially those bearing a boron Lewis acid and a phosphine oxide Lewis base, have become attractive motifs for tunable luminescence properties in materials. However, intramolecular Lewis adducts with Ch­(IV)=O moieties (Ch = chalcogen) as Lewis bases are under-represented in the field. Here, we describe the syntheses of two chalcogen boranes of general formula *o*-(PhCh)­(BMes_2_)­C_6_H_4_ (Ch = S (**1**), Se (**2**)) and their conversions into the corresponding chalcogen-oxide boranes of general formula *o*-(PhCh=O)­(BMes_2_)­C_6_H_4_ (Ch = S (**3**), Se (**4**)). While both **3** and **4** form inner adducts held by Ch=O→B dative bonds in the solid state and in solution, we examine the bistabilities of these interactions computationally and experimentally. Interestingly, the reaction of **4** and HF·pyridine gives rise to *o*-(SePhMes)­(BF_3_)­C_6_H_4_ (**5**) which shows evidence for F→Se intramolecular chalcogen bonding.

## Graphical abstract

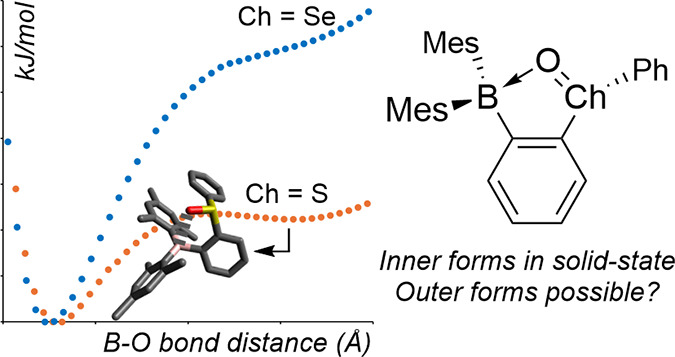

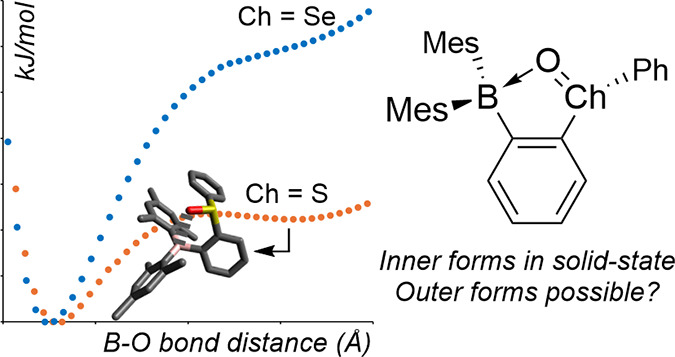

The Gutmann-Beckett method^1,2^ is a hallmark, if imperfect,^3,4^ experiment in main group chemistry that involves the treatment of a Lewis acid with Et_3_P=O. In the case of boron Lewis acids, Et_3_P=O interacts with the empty boron p-orbital to form an intermolecular P=O→B linkage.^5^ Extensions of this experiment are intramolecular Lewis adducts (ILAs) which feature P=Ch→B (Ch = O, S, Se) dative bonds and are typically the products of frustrated Lewis pair chemistry^6–10^ or synthetic curiosities.^11,12^ Wolf and co-workers later made use of this motif with **A** (Figure 1),^13^ whose luminescent properties could be tuned by cleaving the P=O→B dative bond of the closed or inner form^14,15^ in the ground state. Since then, other P=Ch→B ILAs have also been reported with tunable properties resulting from the bistability of the open and closed forms,^16^ including **B** which accesses the open form upon photoexcitation.^17^

**Figure 1:**
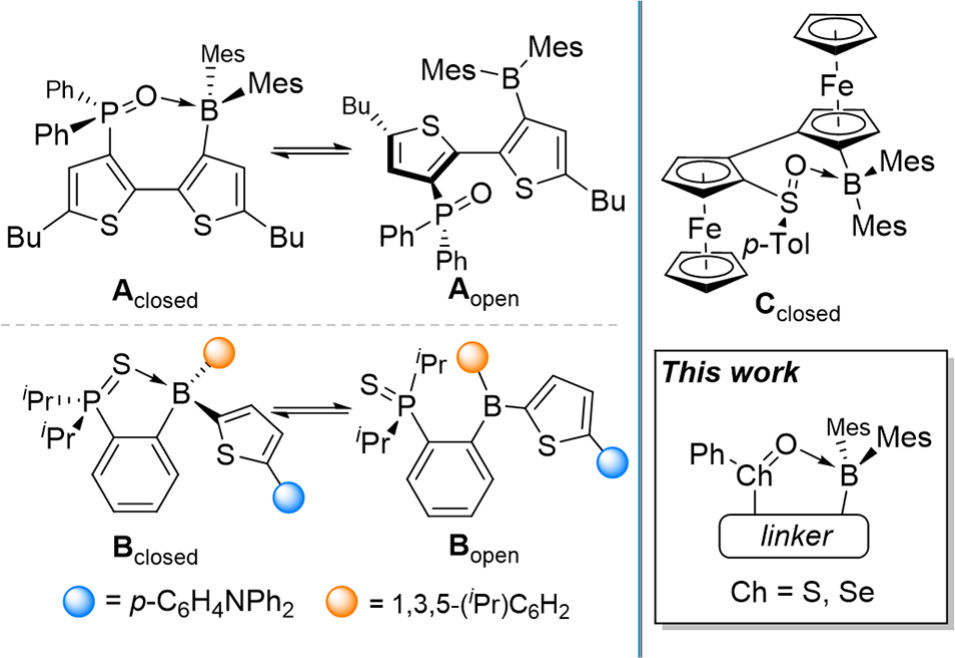
Important precedents of main group oxide borane ILAs and the investigative framework of this study.

By contrast, boron-based ILAs that contain chalcogen oxides as intramolecular donors are comparatively poorly explored.^18^ For instance, while Jäkle and co-workers have crystallized **C** in the inner form, ^11^B NMR spectroscopy indicates that this S=O→B interaction breaks in solution.^19^ ILAs featuring R_2_Se=O→B interactions are completely absent from the literature. With the paucity of fundamental studies into these systems as a backdrop, we report the synthesis and characterization of sulfoxide- and selenoxide-borane ILAs, and we explore their bistabilities.

While sulfur borane **1** is known (), *o*-(PhSe)­C_6_H_4_Br^20^ was treated with one equiv of *^n^* BuLi in THF at −78 °C followed by the addition of Mes_2_BF to afford selenium borane **2** (Scheme 1) as a pale-colored powder. This compound has been characterized by multinuclear NMR spectroscopy and X-ray crystallography (). At 3.283(4) Å, the selenium and boron centers are well-below the sum of their van der Waals radii (∑_vdW_(Se–B): 3.73 Å).^21^ Nevertheless, a broad resonance at 73.4 ppm present in the ^11^B­{^1^H} NMR spectrum and the sum of the C_aryl_–B-C_aryl_ angles being nearly 360° clearly indicate an uncompromised triarylborane.

**Scheme 1:**
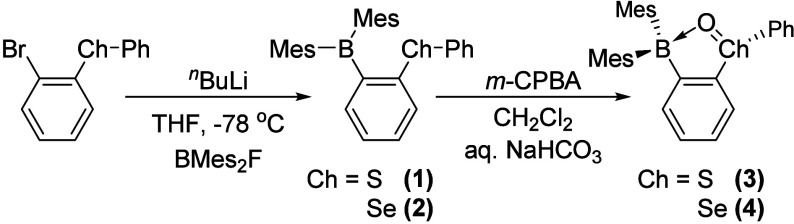
Synthesis of **1**-**4**

Treatment of **1** and **2** with 1.1 equiv of 3-chloroperoxybenzoic acid (*m*-CPBA) in CH_2_Cl_2_ yielded colorless solids **3** and **4**, respectively, following workup with saturated aq. NaHCO_3_. The resulting ^1^H NMR spectra of **3** and **4** display complete desymmetrization of the mesityl-based resonances.^22^ Sharp peaks in their ^11^B­{^1^H} NMR spectra can also be found at 18.2 ppm in CD_3_CN for **3** and 14.7 ppm in acetone-*d*_6_ for **4**, which are indicative of a tetracoordinate boron arising from Ch=O→BMes_2_ interactions. In the case of **4**, the successful oxidation of the selenium center was also confirmed by a downfield shift in the ^77^Se­{^1^H} NMR resonance from 431.8 ppm for **2** in CD_2_Cl_2_ to 943.3 ppm for **4** in acetone-*d*_6_.

The formation of the expected products was confirmed by the elucidation of their solid-state structures. To begin, the BMes_2_ moiety of **3** pyramidalizes upon interacting with the S=O bond yielding a B–O bond length of 1.651(5) Å (Figure 2). This interaction also lengthens the S=O bond (1.546(2) Å) compared to that of free Ph_2_SO (1.4911(11) Å),^23^ putting it on par with that found in **C** (1.556(2) Å).^19^ Infrared (IR) spectroscopy performed on crystals of **3** confirm the decrease S–O double bonding with the S–O stretch at 910 cm^–1^ being red-shifted compared to that reported for free Ph_2_SO (1041 cm^–1^).^24^ The solid-state structure of **4** is quite similar to that of **3**, providing a rare example of an ILA with the selenoxide as the Lewis base (Figure 2).^25,26^ Coordination of the Se=O moiety to the boron center also lengthens the Se=O bond to 1.719(2) Å compared to 1.6756(16) Å in Me_2_SeO,^27^ approaching the Se–O distance found in protonated diarylselenoxides (1.74–1.76 Å).^28,29^ The resulting B–O linkage of 1.632(4) Å is also slightly shorter than that found in **3**, which we attribute to the higher basicity of the selenoxide moiety.^30^ We were also able to locate the Se–O bond stretch at 734 cm^–1^ by IR spectroscopy on a solid-state sample () which is red-shifted compared to Me_2_SeO (820 cm^–1^).^31^

**Figure 2:**
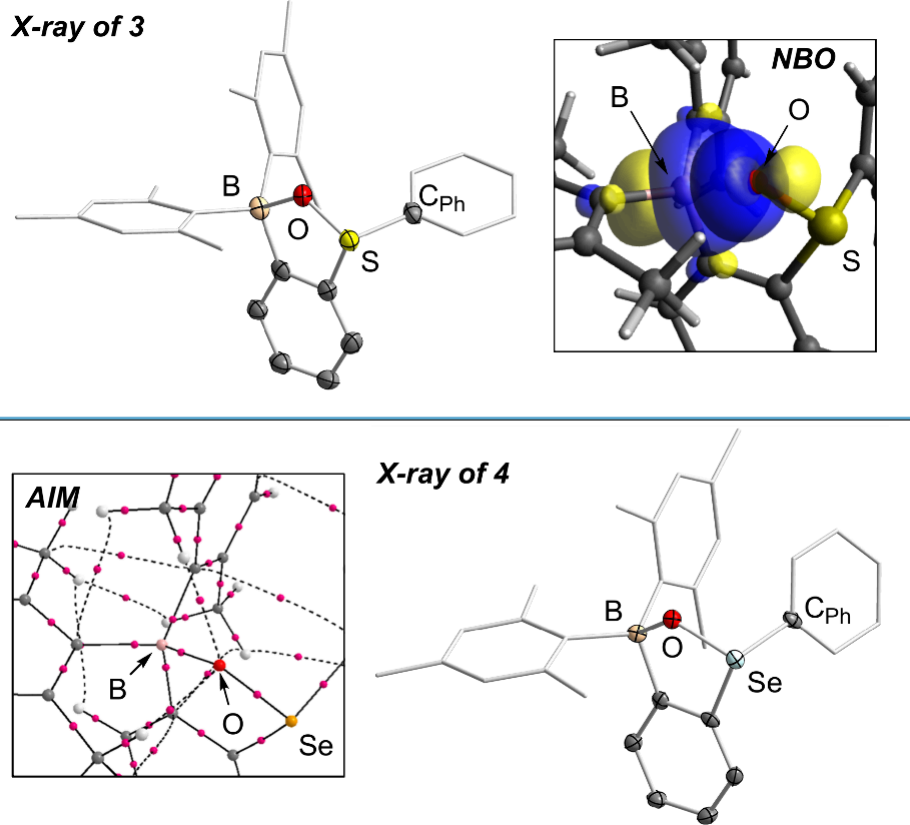
Top: solid-state structure of **3** and representative NBOs involved in the lp­(O)→p­(B) interaction present in **3** (isovalue: 0.05). Hydrogen atoms have been omitted for clarity. Bottom: solid-state structure of **4** and AIM plot showing the bond path between the O and B atoms. Hydrogen atoms have been omitted for clarity.

We then conducted computational analyses on **3** and **4** via density functional theory (DFT) methods to shed further light on the Ch=O→B interactions. Natural Bond Orbital (NBO) analyses were performed on the optimized structures of **3** and **4**, which identified these linkages as strong lp­(O)→p­(B) donor–acceptor interactions (Figure 2 and ). Atoms in molecules (AIM) analyses were also implemented on **3** and **4**, which indicated that the Ch=O→B interactions are quite strong, with high ρ­(r) values of 0.0919 and 0.1026 e^–^ Bohr^–3^ at the localized bond critical points (BCPs), respectively (Figure 2 and ). This greater electron density in **4** again points to the higher basicity of the selenoixde moiety.

In line with our recent study on the inner and outer forms of a carbenium-phosphine oxide-based ILA,^15^ we then became curious if the outer forms of **3** or **4** were accessible in the ground state. To that end, we subjected both **3** and **4** to relaxed scan calculations that incrementally increase the distance between the Lewis opposite moieties. In the case of **3**, the inner form (**3**_**I**_) predominates, in accordance with our characterization of the molecule, but the outer form (**3**_**O**_) was also identified as a shallow yet defined well on the potential energy surface (Figure 3). This afforded us a minimum structure at which the B···O bond is significantly elongated to 2.847 Å. This bond length can be compared with the B···N distance computed for the outer form of nitrile–borane adducts^32,33^ such as that of HCN→BCl_3_ (2.817 Å).^34^ We also note a similarity with the P=O···B distance found in the solid-state structure of *o*-(Ph_2_P=O)­(BPin)­C_6_H_4_ (2.7968(15) Å).^35^ By contrast, no second well-defined minimum was found for **4**, and the energy penalty incurred to reach its presumed outer region is far more substantial than that found for **3**. Indeed, we propose that this is a result of the greater Lewis basicity of the selenoxide moiety overwhelmingly favoring the dative bond found in the **4**_**I**_ form.

**Figure 3:**
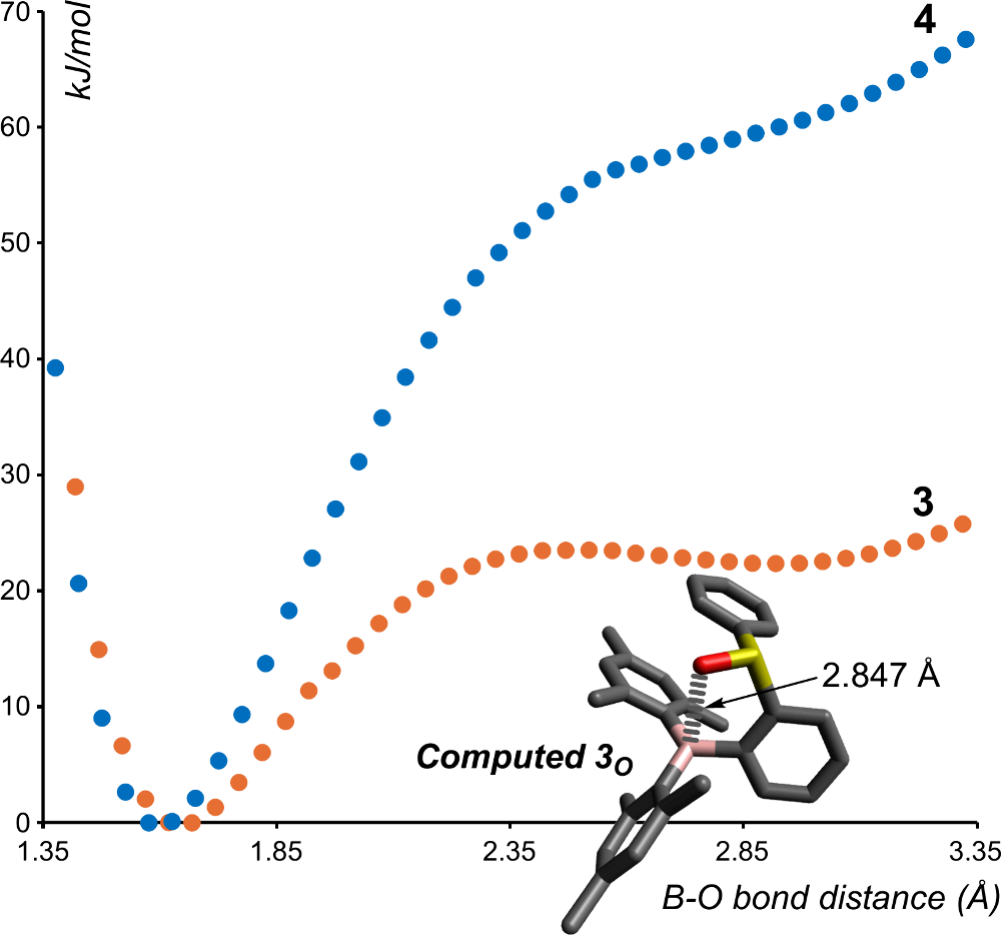
Relaxed potential energy surface of **3** (orange dots) and **4** (blue dots) by elongation of the B–O distance showing the existence of two minima for **3**. The energies in kJ/mol are relative to those found for optimized **3**_**I**_ and **4**_**I**_. Inset shows the optimized structure of **3**_**O**_ and its long B–O bond.

Lastly, we became eager to find conditions that could cleanly cleave the Ch=O→B dative bonds. In an attempt to generate their fluoroborate salts, **3** and **4** were treated with excess TBAF·3 H_2_O, though this resulted in complicated multinuclear NMR spectra of unidentified products. Thus, to capture potential decomposition products as trifluoroborate salts, we subjected both compounds to excess HF·pyridine in CH_2_Cl_2_.^36,37^ While aqueous workup of **3** provided an intractable mixture of unidentifiable products, the reaction with **4** reproducibly afforded a colorless powder with clean NMR spectra. Curiously, while the ^11^B­{^1^H} NMR spectrum of this material clearly shows a quartet belonging to a trifluoroborate moiety, the ^1^H NMR spectrum reveals that a mesityl group persisted throughout the course of the reaction. Of particular note is the ^77^Se­{^1^H} NMR of **5** which reveals a quartet at 490.9 ppm with a *J*_Se–F_ coupling constant of 50.5 Hz, ruling out the presence of a Ar_2_SeF_2_ moiety^29^ but potentially indicating weak Se–F coupling.

Indeed, X-ray diffractometry performed on a crystalline sample of this product revealed the formation of a zwitterionic aryltrifluoroborate with an *o*-triarylselenonium cation as its charge-opposite partner (**5**, Figure 4). Given the dehydration of the selenoxide moiety and the known reactivity of diaryltellurium difluorides toward arylboronates,^38,39^ we propose that the Se=O→B breaks upon protonation, the Se­(IV) center is difluorinated, and the mesityl group is intramolecularly transferred from the boron center to form the selenonium cation (). Both crystallized enantiomers in the unit cell of **5** situate at least one of their fluorides near the selenonium center (Se_1_–F_1_: 2.819(4) Å, Se_2_–F_4_: 2.871(5) Å) at a distance below the sum of these elements’ van der Waals radii (∑_vdW_(Se,F) = 3.28 Å).^21^ Taken with the nearly linear F–Se–C_Mes_ angles formed in both cases (F_1_–Se_1_-C_13_: 174.9(2)°, F_4_–Se_2_-C_34_: 173.0(2)°) and considering the NMR data, we contend that **5** exhibits intramolecular F→Se chalcogen bonding.^40^ We note also that while this compound bears similarity to the previously described *o*-(SMe_2_)­(BF_3_)­C_6_H_4_,^41^ we are not aware of a similar molecular motif in the literature.

**Figure 4:**
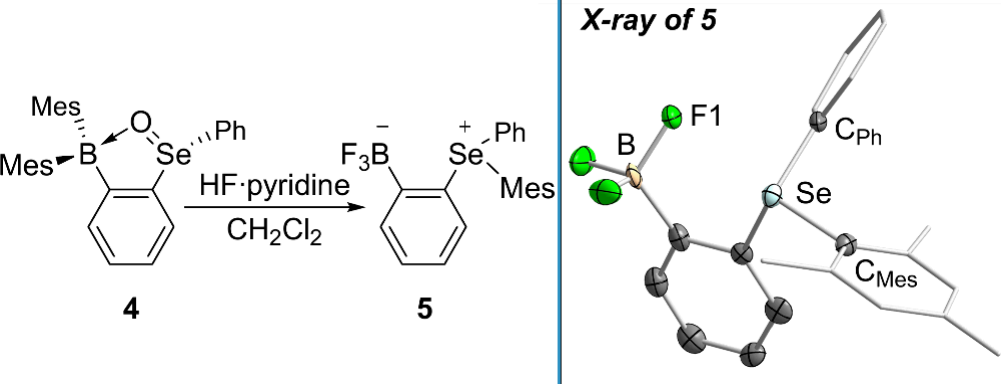
Left: synthesis of **5**. Right: solid-state structure of **5**. Only one of the two independent molecules found in the cell is shown. Pertinent metric information can be found in the text.

In summary, we report the formation of chalcogen oxide boranes, which form strong Ch=O→B dative bonds in the crystal and in solution. Inner-outer isomerism was also probed computationally, and reactivity toward an HF source was investigated. This study highlights new potential platforms for ILAs that may be useful for new stimulus-responsive luminescent materials and tunable photoredox catalysis.

## Supplementary Material





## Notes

Additional experimental and computational details (PDF)
Optimized structures in xyz format (XYZ)
