# Supplementary material for: Flash Communication: On the Bistabilities and Reactivities of ortho-Phenylene Compounds with Ch=O→B Interactions
Source: Organometallics. 2025 Aug 5;44(16):1751–5. doi: 10.1021/acs.organomet.5c00229 (PMC12381915; doi:10.1021/acs.organomet.5c00229)
Supplement: Supplementary file 1 [file om5c00229_si_001.pdf]

Flash Communication: On the bistabilities and reactivities of *ortho*-phenylene compounds with Ch=O→B interactions

Brendan L. Murphy and François P. Gabbaï*

Department of Chemistry, Texas A&M University, College Station, Texas 77843-3255, United States

**Corresponding author email: francois@tamu.edu*

Supporting Information

This PDF file includes

Contents:

1. Synthesis and characterization

1.1. General considerations

1.2. Synthetic procedures & NMR spectra

2. Computational Details

2.1. General considerations

2.2. NBO output

2.3. AIM output

3. Mechanism proposal for formation of 5

1. Synthesis and characterization

1.1. General considerations

1[1] and *o*-(PhSe)(Br)C_6H_4[2] were prepared according to literature procedures. We note that **1** was not fully characterized, and we provide the associated data on pages S3-S7. The following materials were purchased from their associated suppliers and used without further purification:

- Acros Organics: 3-chloroperoxybenzoic acid (70-75% balance 3-chlorobenzoic acid and water; *m*-CPBA)
- Alfa-Aesar: Hydrogen fluoride pyridine complex (ca. 70% HF; HF·pyridine)
- TCI America: dimesitylboron fluoride ($BMes_2F$)

Solvents were dried over Na/K (THF) or CaH_2 (CH_2Cl_2) and were distilled prior to use. NMR spectra were recorded at room temperature using a Bruker Avance 500 spectrometer (499.58 MHz for ^{1}H, 125.63 MHz for ^{13}C), a Bruker Ascend 500 NMR spectrometer (500.13 MHz for ^{1}H, 125.63 MHz for ^{13}C, 160.46 MHz for ^{11}B, 470.55 MHz for ^{19}F), or a Bruker Ascend 400 NMR spectrometer (400.09 MHz for ^{1}H, 100.61 MHz for ^{13}C, 128.36 MHz for ^{11}B). Chemical shifts are given in ppm and are referenced against residual solvent signals (^{1}H, ^{13}C) or external standards (for ^{11}B: 0.00 ppm for $BF_3 \cdot Et_2O$; for ^{19}F: C_6F_6 at -162.61 ppm versus $CFCl_3$ in CD_2Cl_2;[3] for ^{77}Se: Ph_2Se_2 at 463.0 ppm versus Me_2Se[4]). Elemental analyses (EA) were performed at Atlantic Microlab (Norcross, GA). Mass spectrometry analysis was performed in-house at the Center for Mass Spectrometry using a Thermo-Scientific Q Exactive Focus instrument. Infrared spectra were recorded using a Shimadzu IRAffinity FTIR spectrometry equipped with a Pike MIRacle attenuated total reflectance device.

All crystallographic measurements were performed at 110(1) K using a Bruker D8 QUEST diffractometer (Mo-Kα radiation, λ = 0.71073 Å) or a Rigaku XtaLAB Synergy diffractometer (Cu-Kα radiation, λ = 1.5406 Å). In each case, a specimen of suitable size and quality was selected and mounted onto a nylon loop. Integrated intensity information for each reflection was obtained by reducing the data frames using APEX3[5] or CrysAlis^{Pro}.[6] The semi-empirical methods SADABS[7] and ABSPACK[cite] were used for the absorption corrections in APEX3 and CrysAlis^{Pro}, respectively. The structures were solved by direct methods using ShelXT[8] and refined against F^2 with anisotropic temperature-dependent parameters for all non-hydrogen atoms using ShelXL[9] using Olex2.[10] All H-atoms were geometrically placed and refined using a riding model. Diamond4 was used for final data presentation. **5** displayed evidence of non-merohedral twinning. The major component was identified and the pertinent reflection data was refined against it, leading to an improvement in the quality of the structure. CCDC 2465147-2465151 contains the supplementary crystallographic data for this paper. These data can be obtained free of charge via www.ccdc.cam.ac.uk/data_request/cif, or by emailing data_request@ccdc.cam.ac.uk, or by contacting The Cambridge Crystallographic Data Centre, 12 Union Road, Cambridge CB2 1EZ, UK; fax: +44 1223 336033.

1.2. Synthetic procedures

Synthesis of **1**: **1** was synthesized using previously described procedures. Single crystals were obtained as colorless blocks *via* layering pentane onto a CH_2Cl_2 solution of the compound.

^{1}H NMR (400 MHz, CD_2Cl_2) δ 7.24 (m, 6H, S*Ph* & $C_6\mathit{H}_4$), 7.15 (m, 2H, S*Ph* & $C_6\mathit{H}_4$), 7.07 (d, 1H, J = 7.8 Hz, $C_6\mathit{H}_4$), 6.78 (s, 4H, Mes-C*H*), 2.28 (s, 6H, *p*-Mes-$C\mathit{H}_3$), 2.03 (broad s, 12H, *o*-Mes-$C\mathit{H}_3$).

^{13}C NMR (101 MHz, CD_2Cl_2) δ 150.4 (broad s), 143.4 (broad s), 142.4 (s), 141.23 (s), 139.9 (s), 136.6 (s), 134.7 (s), 132.8 (s), 131.3 (s), 130.9 (s), 129.6 (s), 128.8 (s), 127.8 (s), 126.5 (s), 23.4 (s), 21.6 (s).

^{11}B{^{1}H} NMR (128 MHz, CD_2Cl_2) δ 74.2 (broad s).

Elemental analysis calculated for $C_{30}H_{31}BS$: C 82.94, H 7.19; found: C 81.77, H 7.36.

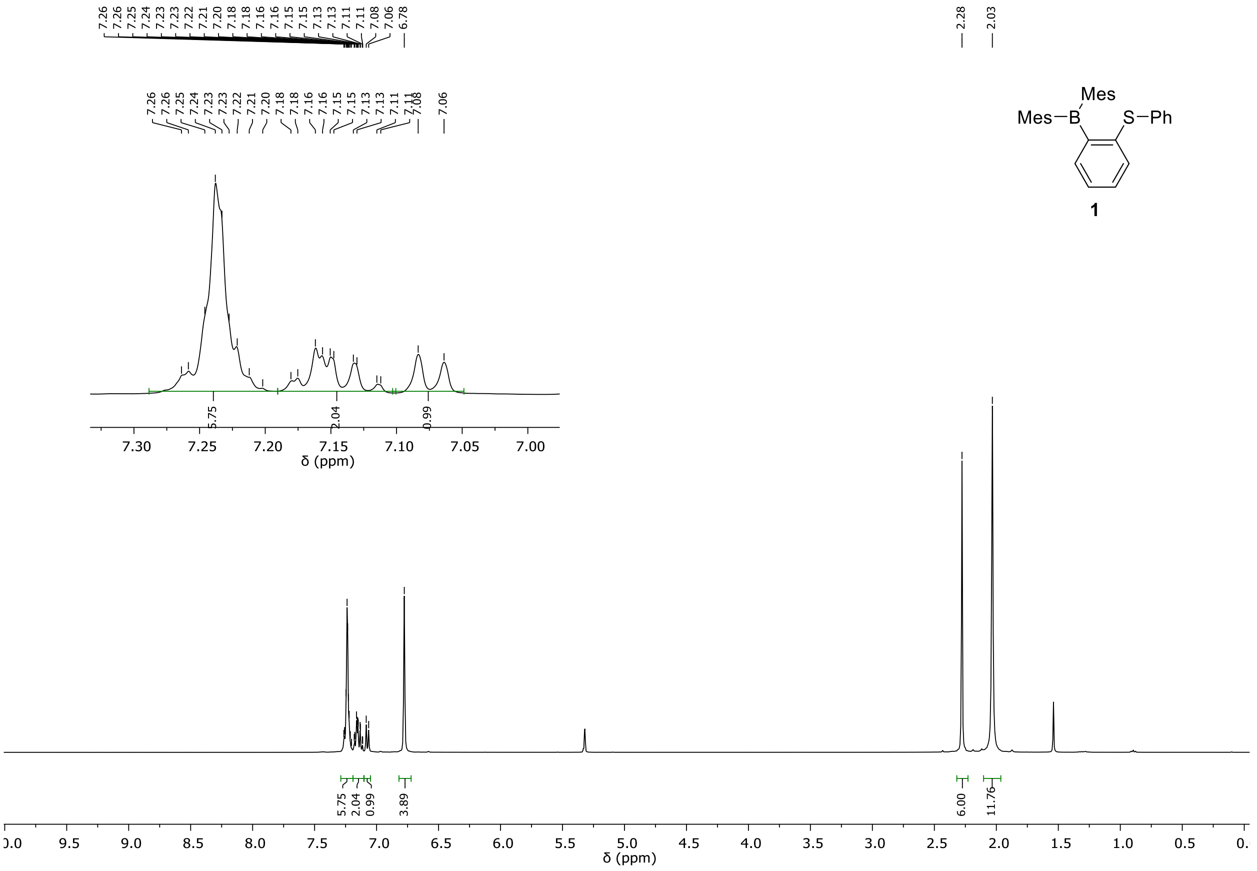

Figure S1. ^1H NMR (CD_2Cl_2, 400 MHz) spectrum of **1**.

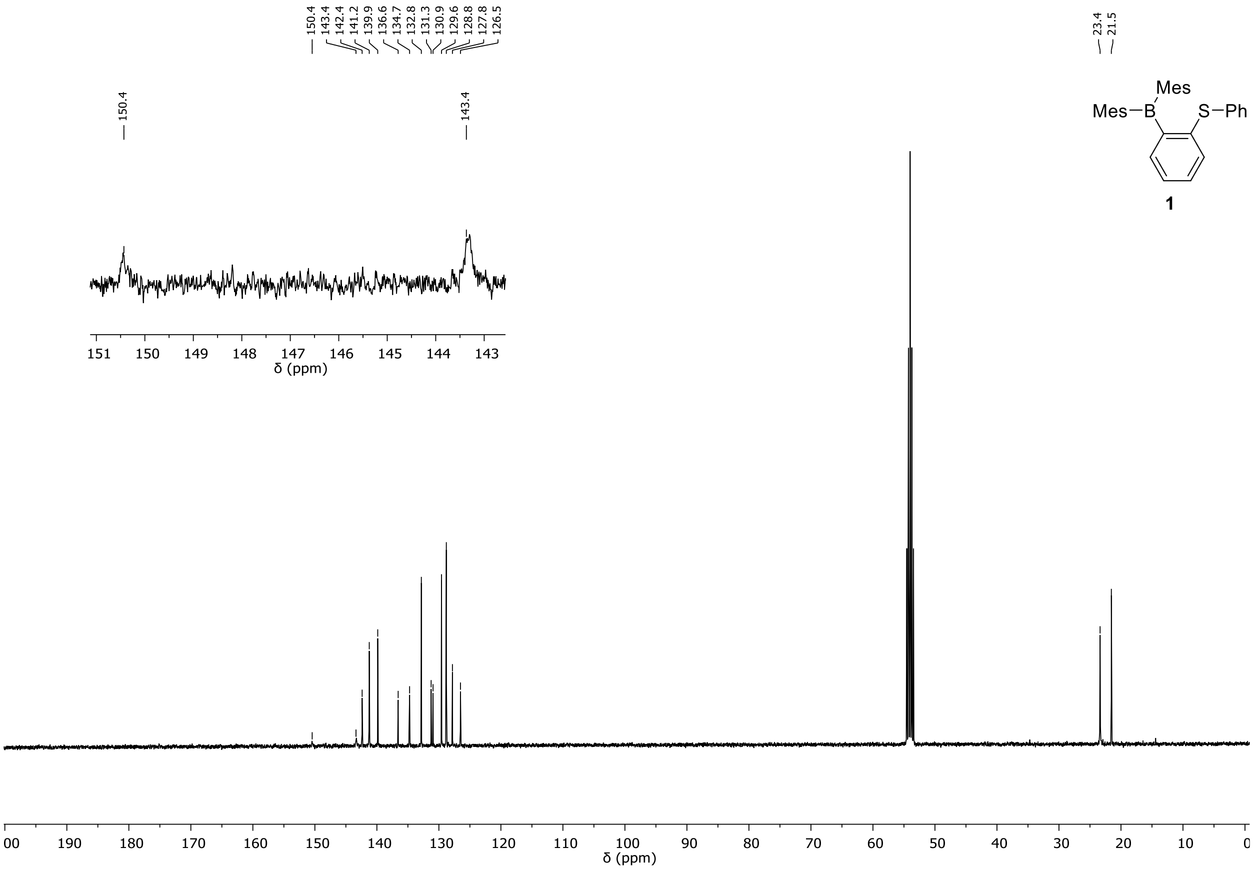

Figure S2. ^{13}C NMR (CD_2Cl_2, 100 MHz) spectrum of **1**.

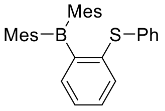

1

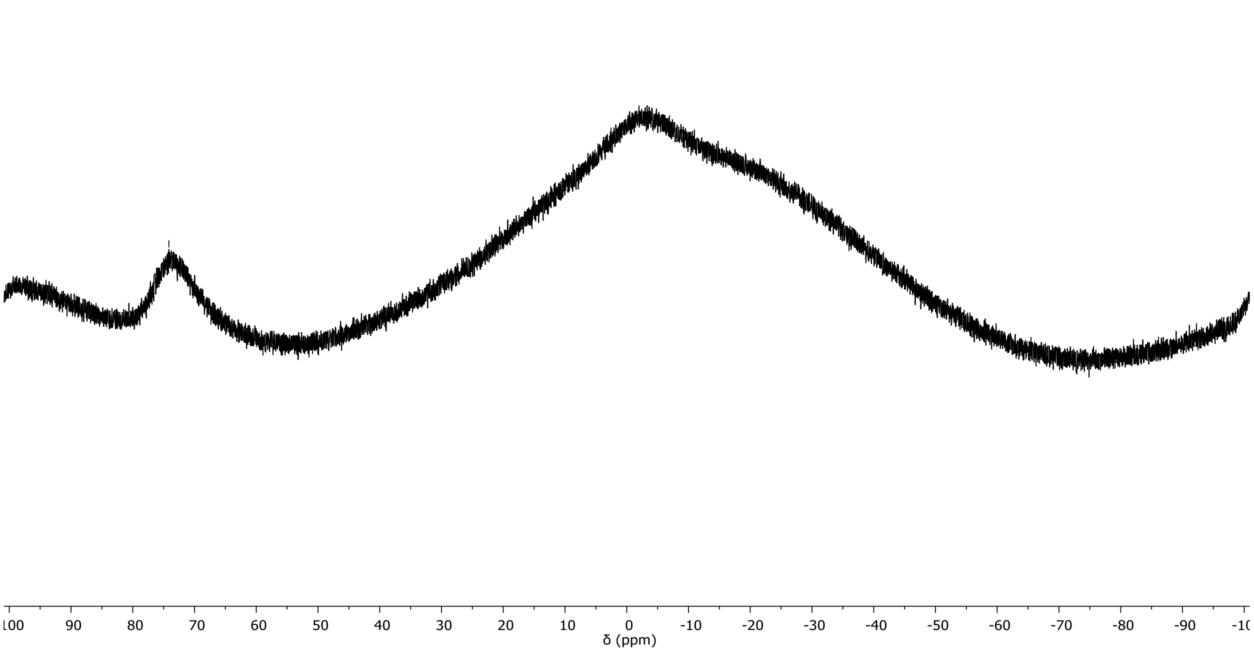

Figure S3. $^{11}B\{^{1}H\}$ NMR (CD_2Cl_2, 128 MHz) spectrum of **1**.

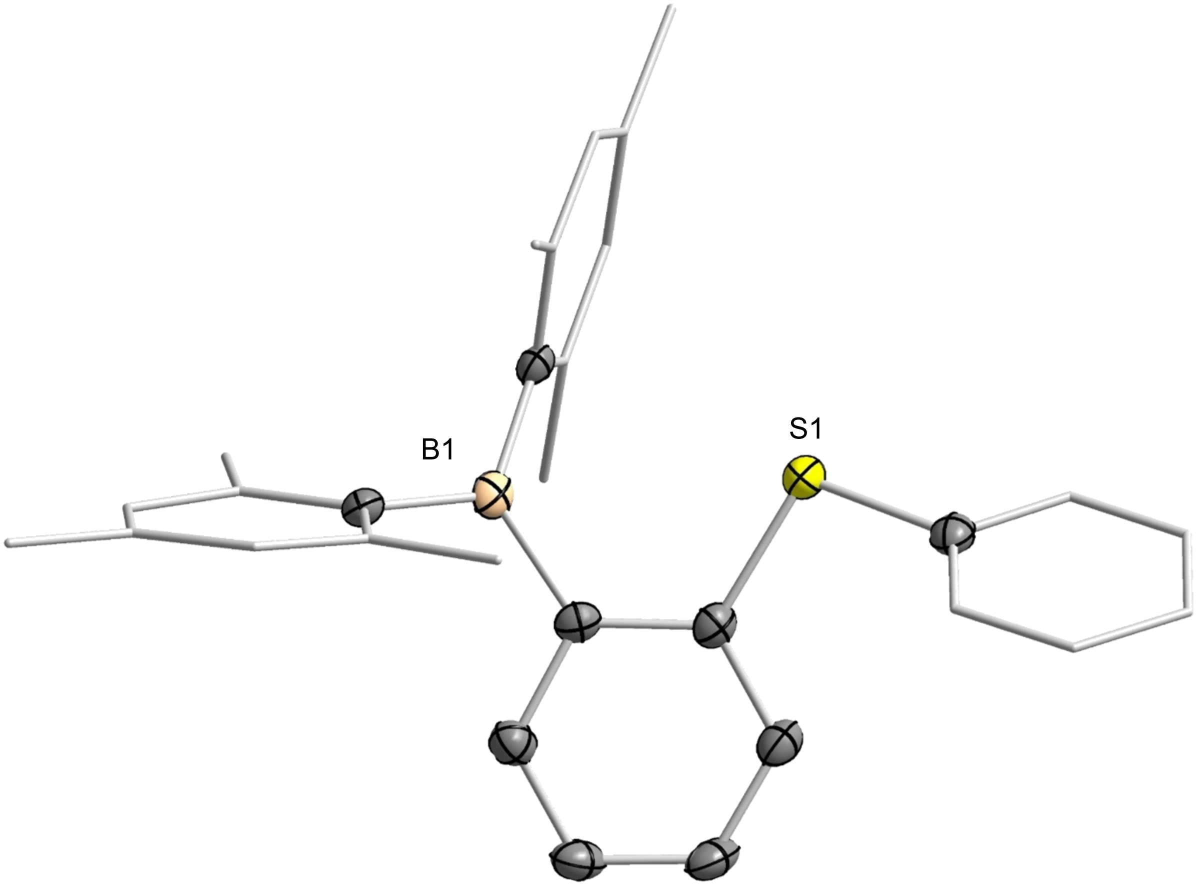

Figure S4. Solid-state structure of **1**. Hydrogen atoms are omitted for clarity.

Synthesis of **2**: A solution of *o*-(PhSe)(Br)C_6H_4 (1.00 g, 3.20 mmol) in THF (20 mL) was cooled to -78 °C, to which ^{n}BuLi (2.5 M in hexanes, 1.4 mL, 3.50 mmol) was added dropwise. After stirring for 1 h at -78 °C, a solution of $BMes_2F$ (0.95 g, 3.54 mmol) in THF (10 mL) was added *via* canula. The resulting solution was warmed to room temperature and stirred overnight. Solvent was then removed *in vacuo*, the resulting solid was taken up in CH_2Cl_2 (~50 mL) and filtered over Celite. The solution was concentrated *in vacuo* and a pale-colored solid was precipitated following the addition of heptane (~25 mL). The solid was collected by filtration and washed with heptane (3 x 15 mL). Yield: 0.78 g (51%, 1.62 mmol). Single crystals were obtained as colorless blocks *via* evaporation of a CH_2Cl_2:pentane (~1:20, v/v) solution of the compound.

^{1}H NMR (500 MHz, CD_2Cl_2) δ 7.42 (m, 2H, *o*-Se*Ph*), 7.29-7.23 (m, 3H, *p*-Se*Ph* & C_6H_4), 7.16-7.10 (m, 4H, *m*-Se*Ph* & C_6H_4), 6.81 (s, 4H, Mes-C*H*), 2.30 (s, 6H, *p*-Mes-CH_3), 2.05 (broad s, 12H, *o*-Mes-CH_3).

^{13}C NMR (126 MHz, CD_2Cl_2) δ 151.2 (broad s), 143.2 (broad s), 141.6 (s), 140.2 (s), 139.9 (s), 135.5 (s), 135.3 (s), 132.3 (s), 132.2 (s), 131.6 (s), 129.8 (s), 128.3 (s), 126.6 (s), 23.4 (broad s), 21.1 (s).

^{11}B{^{1}H} NMR (128 MHz, CD_2Cl_2) δ 73.4 (broad s).

^{77}Se{^{1}H} NMR (76 MHz, CD_2Cl_2) δ 431.8 (s).

Elemental analysis calculated for $C_{30}H_{31}BSe$: C 74.86, H 6.49; found: C 74.74, H 6.66.

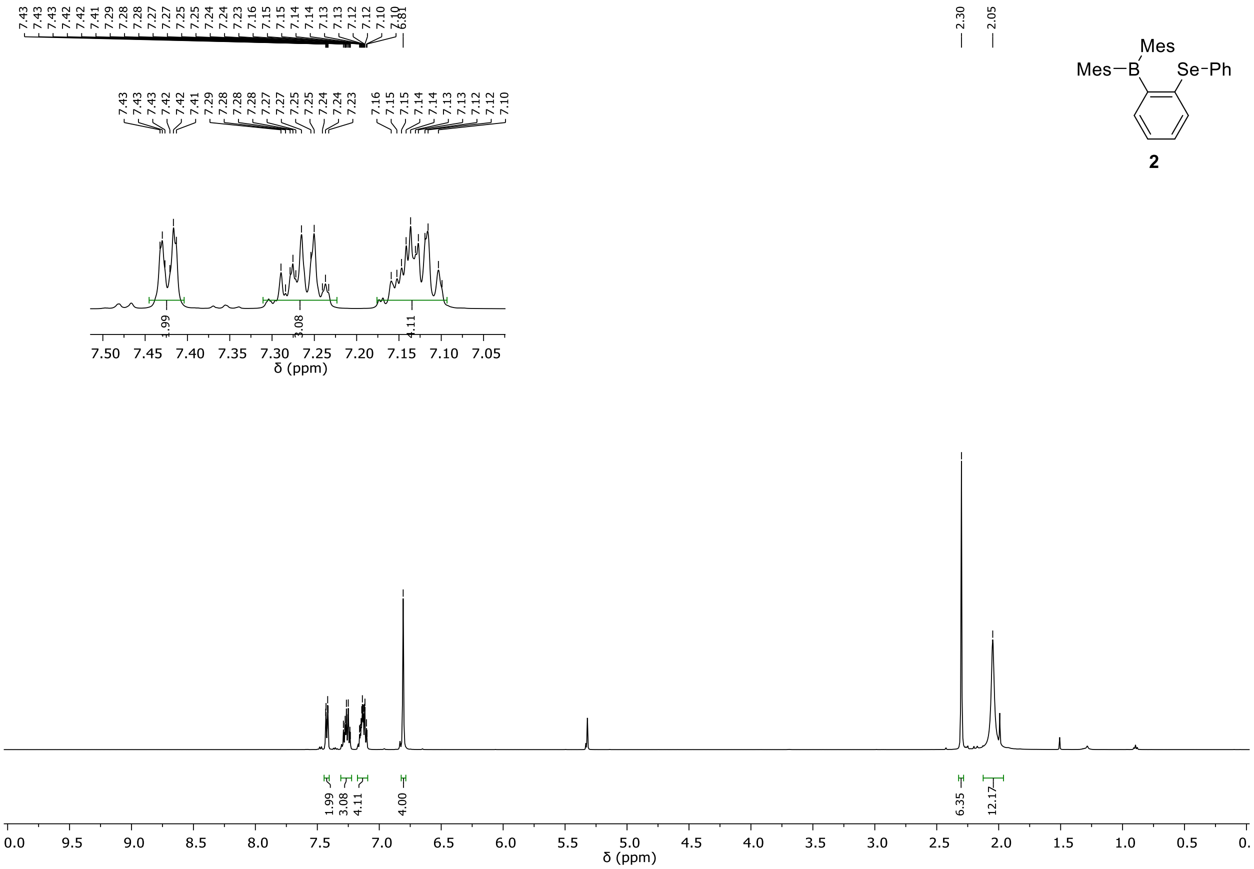

Figure S5. ^{1}H NMR (CD_2Cl_2, 500 MHz) spectrum of **2**.

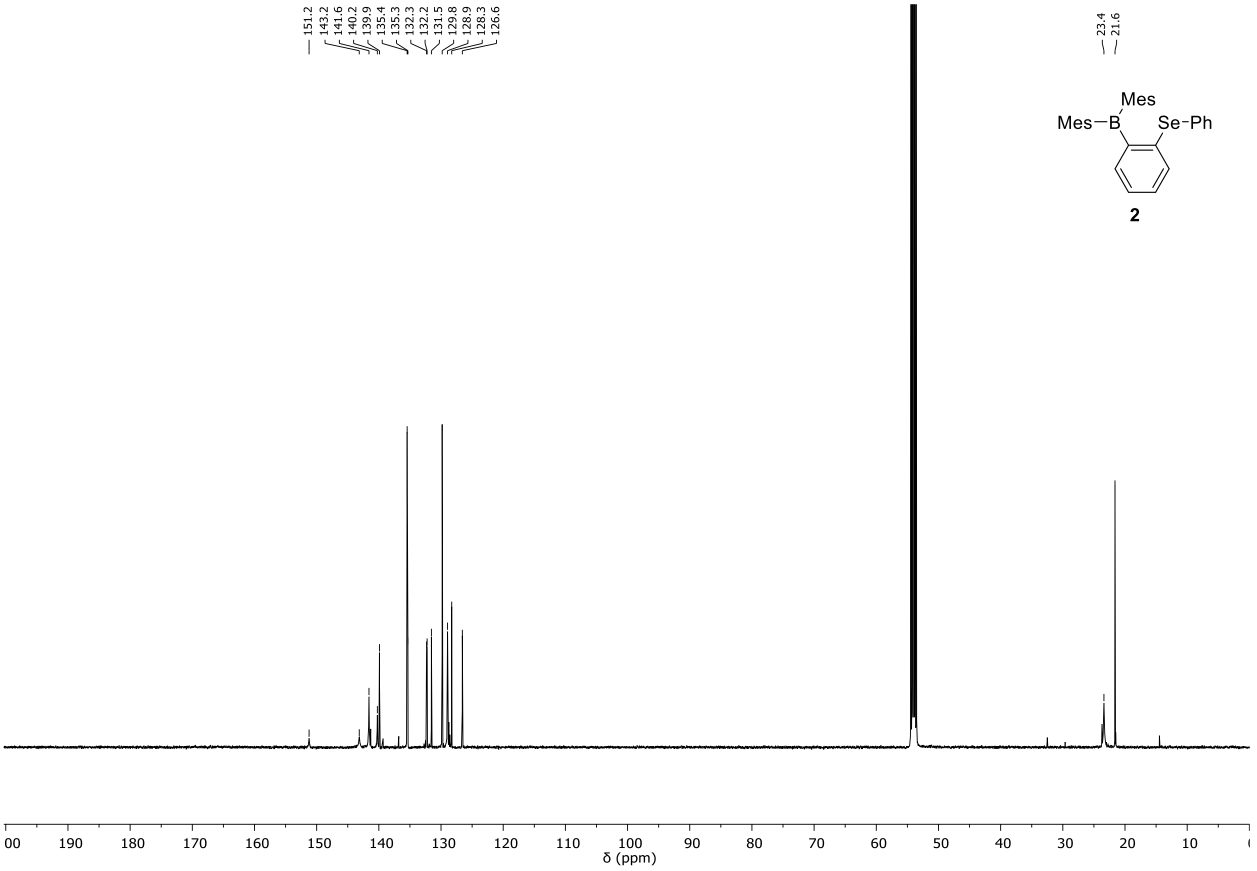

Figure S6. ^{13}C NMR (CD_2Cl_2, 126 MHz) spectrum of **2**.

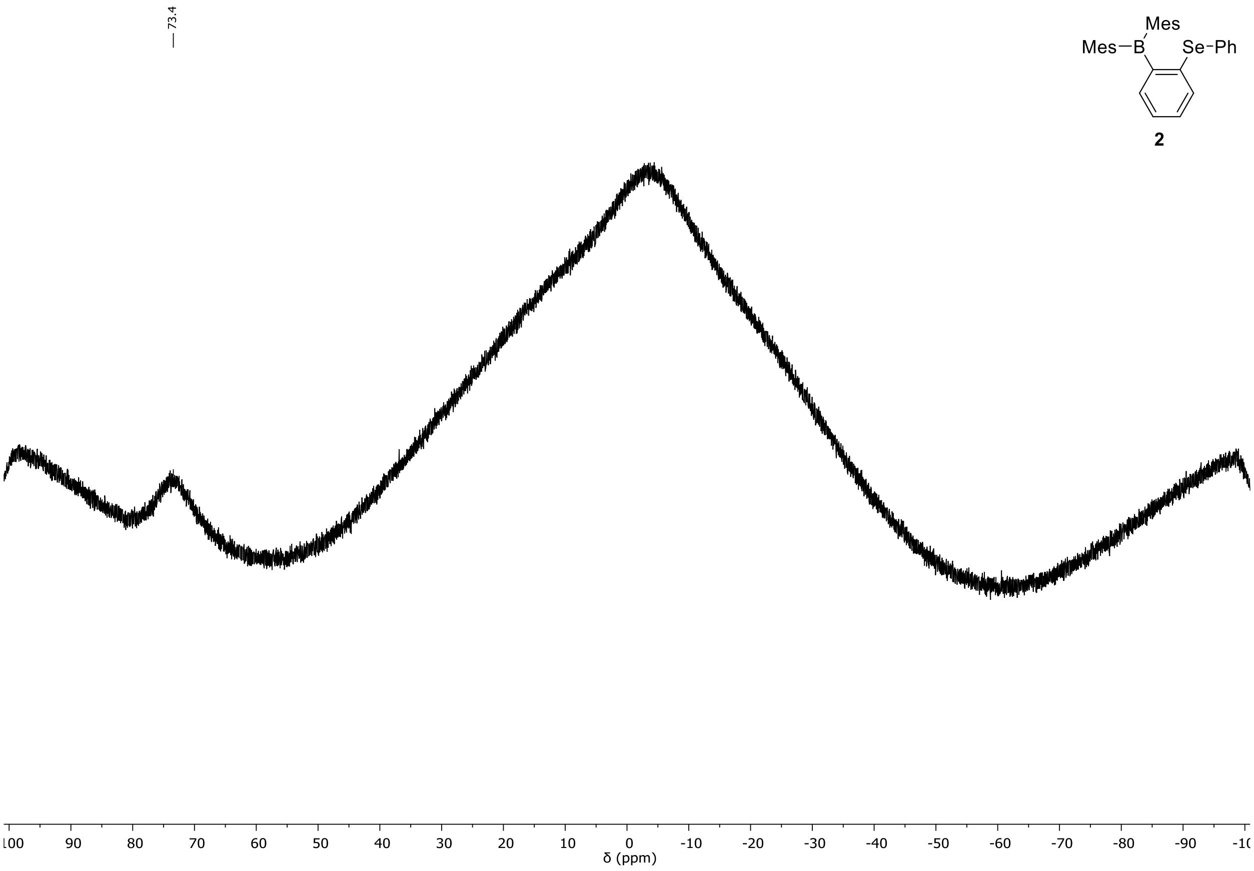

2

Figure S7. $^{11}B\{^{1}H\}$ NMR (CD_2Cl_2, 128 MHz) spectrum of **2**.

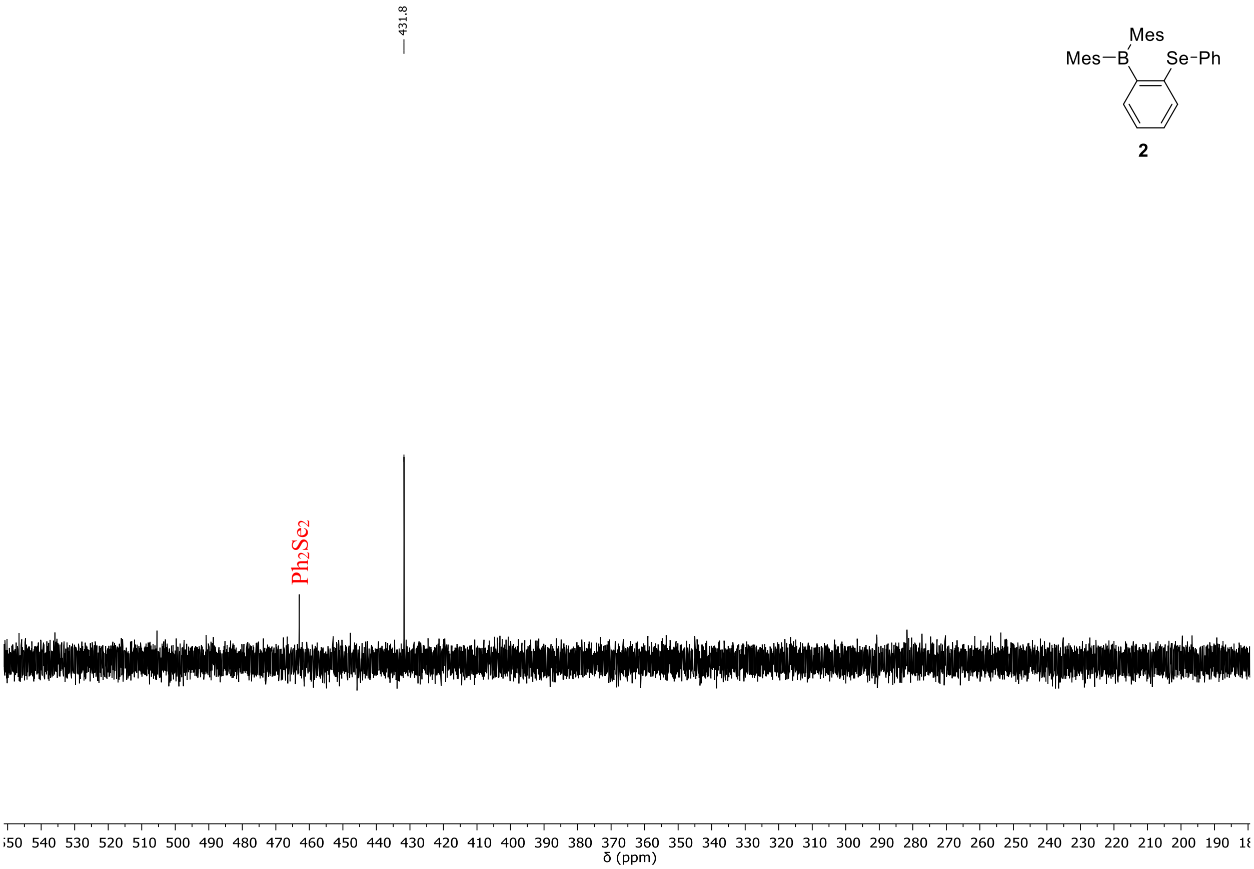

2

Figure S8. $^{77}Se\{^1H\}$ NMR (CD_2Cl_2, 76 MHz) spectrum of **2**.

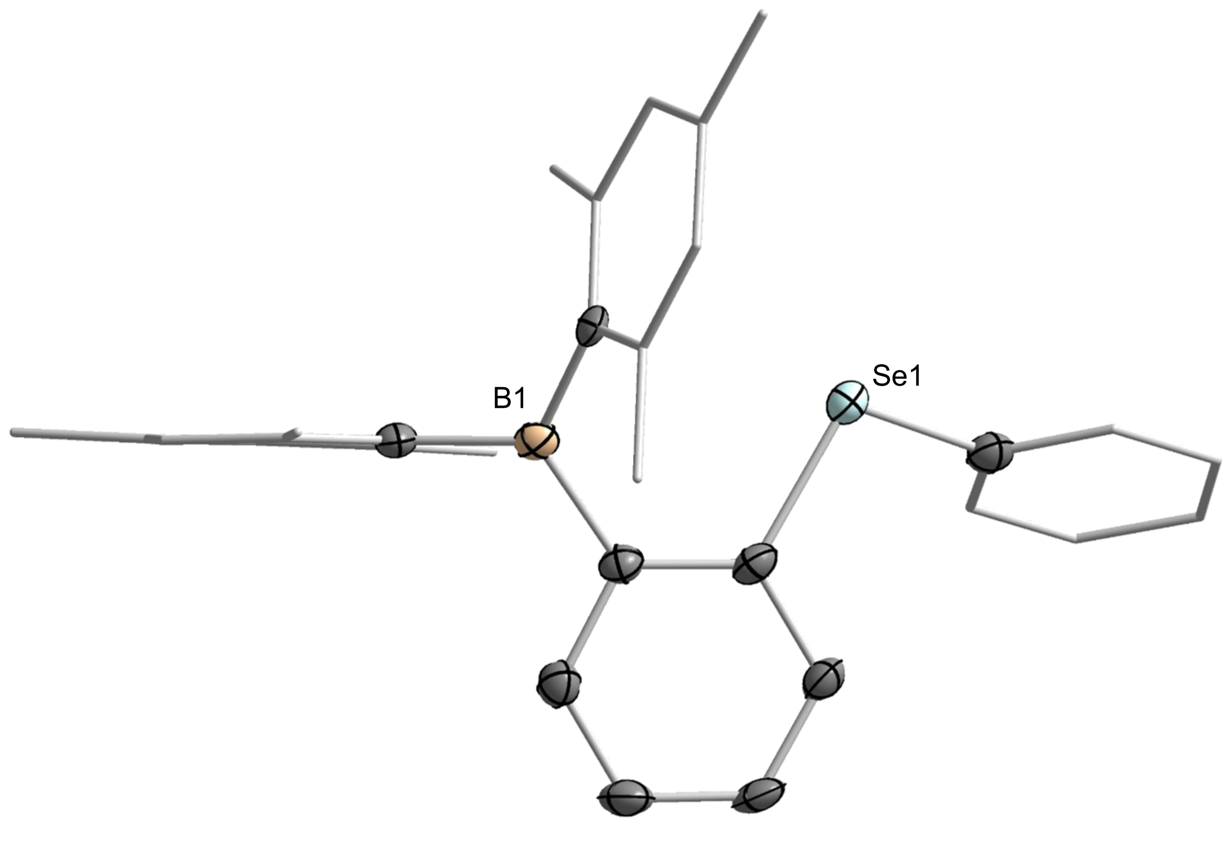

Figure S9. Solid-state structure of **2**. Hydrogen atoms are omitted for clarity.

Synthesis of **3**: A solution of **1** (150.0 mg, 0.35 mmol) in CH_2Cl_2 (20 mL) was cooled to 0 °C. *m*-CPBA (85.0 mg, 0.35 mmol) was added in one portion, and the solution stirred for 30 min. A saturated solution of aq. $NaHCO_3$ (25 mL) was then added and stirred for an additional 30 min. The organic layer was then separated, and the aqueous layer was washed with CH_2Cl_2 (3 x 15 mL). The organic layers were combined, dried over $MgSO_4$, filtered, and solvent was removed *in vacuo*. The resulting residue was triturated with hexanes, and the resulting colorless solid was collected by filtration. Yield: 58.1 mg (37%, 0.13 mmol). Single crystals suitable for X-ray crystallography were obtained *via* evaporation of a CH_2Cl_2 solution of the compound.

^{1}H NMR (500 MHz, CD_3CN) δ 7.68-7.63 (m, 2H, *o*-S*Ph*), 7.49-7.46 (m, 1H, *p*-S*Ph*), 7.43-7.39 (m, 2H, *m*-S*Ph*), 7.38-7.36 (m, 2H, C_6H_4), 7.31-7.28 (m, 2H, C_6H_4), 6.66 (s, 2H, Mes C*H*), 6.56 (s, 2H, Mes C*H*), 2.19 (s, 3H, Mes *p*-CH_3), 2.18 (s, 3H, Mes *p*-CH_3), 2.09 (s, 6H, Mes *o*-CH_3), 1.87 (s, 6H, Mes *o*-CH_3).

^{13}C NMR (126 MHz, CD_3CN) δ 158.74 (br s), 148.61 (br s) 141.93 (s), 140.17 (s), 139.43 (s), 138.46 (s), 135.49 (s), 134.79 (s), 134.31 (s), 133.26 (s), 131.94 (s), 130.51 (s), 130.38 (s), 129.83 (s), 128.36 (s), 127.13 (s), 123.77 (s), 25.53 (s, Mes *o*-CH_3), 24.78 (s, Mes *o*-CH_3), 20.86 (s, Mes *p*-CH_3), 20.72 (s, Mes *p*-CH_3). Note: one aryl C resonance could not be found, likely due to signal broadening and/or incidental overlap.

^{11}B{^{1}H} NMR (128 MHz, CD_3CN) δ 17.6 (s).

Elemental analysis calculated for $C_{30}H_{31}BOS$: C 79.99, H 6.94; found: C 79.85, H 7.01.

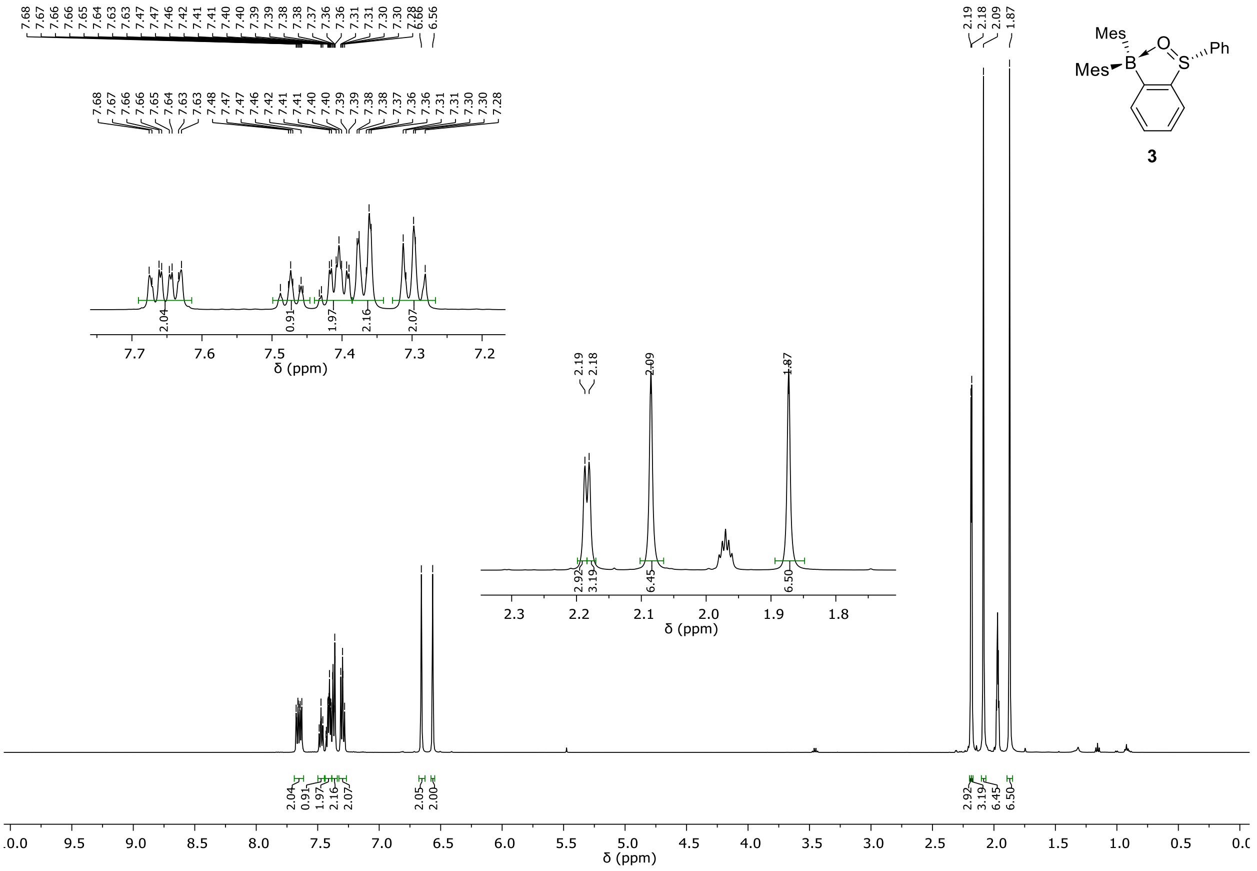

Figure S10. ^{1}H NMR (CD_3CN, 500 MHz) spectrum of **3**.

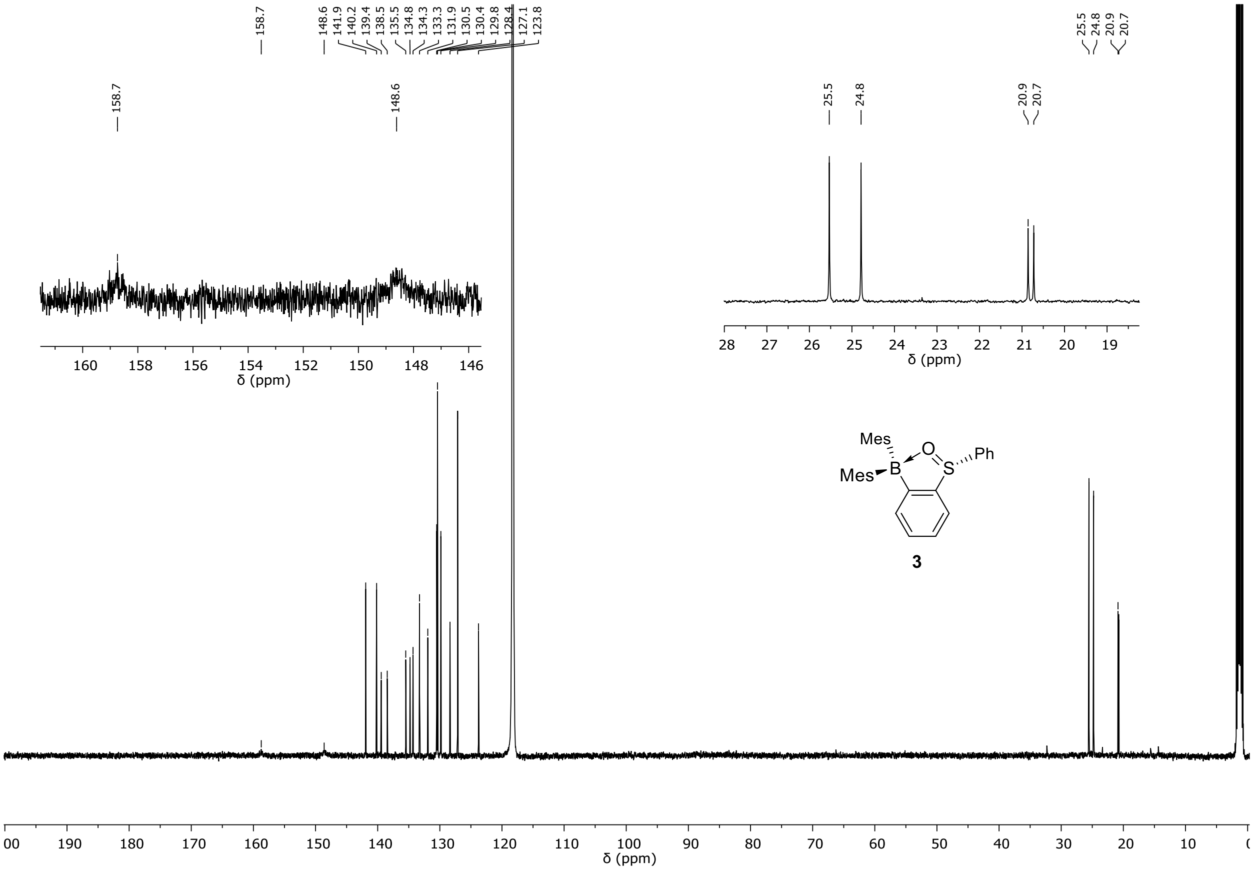

Figure S11. ^{13}C NMR (CD_3CN, 126 MHz) spectrum of **3**. Solvent peaks have been truncated for clarity.

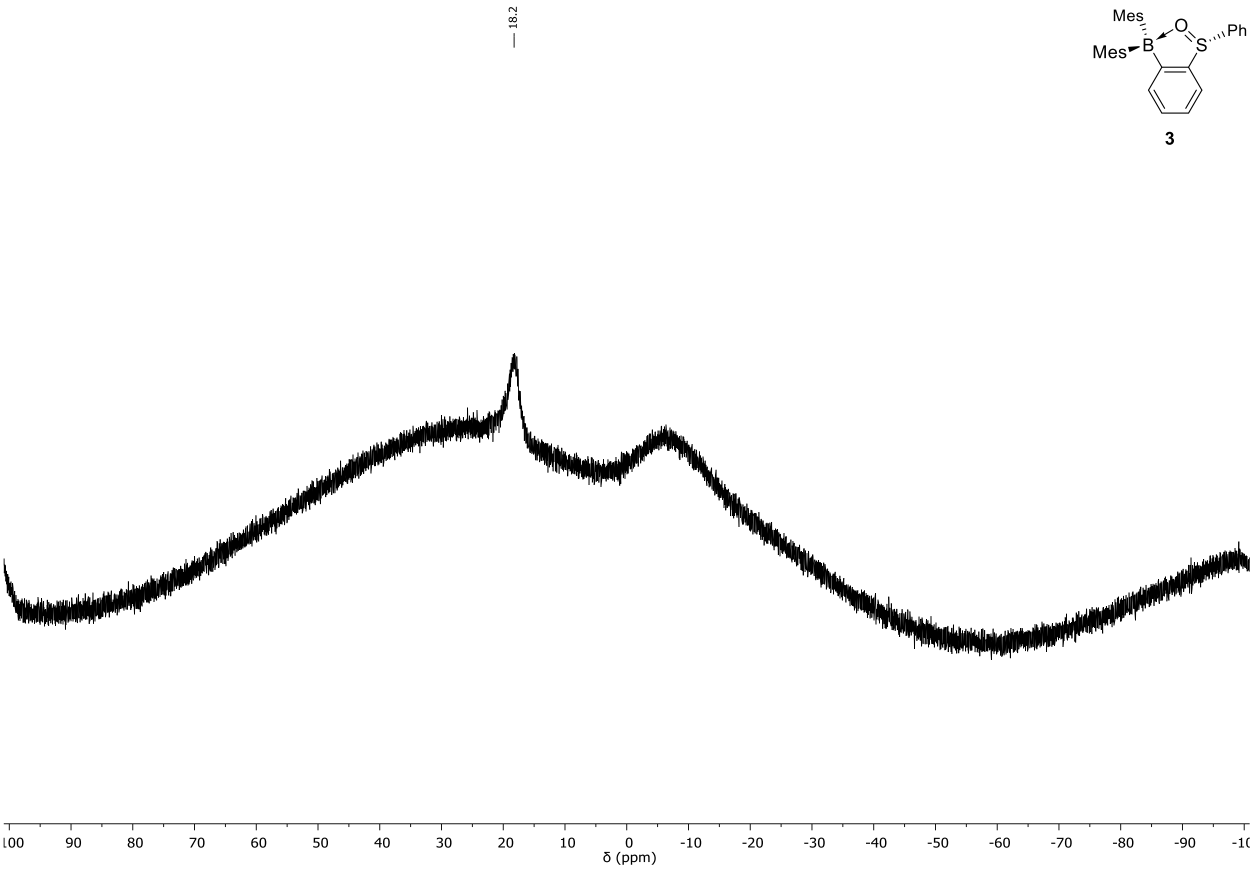

Figure S12. $^{11}B\{^{1}H\}$ NMR (CD_3CN, 128 MHz) spectrum of **3**.

Synthesis of **4**: **2** (200.0 mg, 0.42 mmol) in CH_2Cl_2 (20 mL) was cooled to 0 °C. *m*-CPBA (113.0 mg, 0.47 mmol) was added in one portion, and the solution stirred for 30 min. A saturated solution of aq. $NaHCO_3$ (25 mL) was then added and stirred for an additional 30 min. The organic layer was then separated, and the aqueous layer was washed with CH_2Cl_2 (3 x 15 mL). The organic layers were combined, dried over $MgSO_4$, filtered, and solvent was removed *in vacuo* to yield a colorless solid that was collected on a frit. Yield: 87.4 mg (43%, 0.18 mmol). Single crystals suitable for X-ray crystallography were grown as colorless blocks from evaporation of a $CDCl_3$:Et_2O solution of the compound.

^{1}H NMR (500 MHz, acetone-d_6) δ 7.97 (m, 1H, *p*-Se*Ph*), 7.49 (dd, 1H, *J* = 6.9 Hz & 1.6 Hz, C_6H_4), 7.42 (m, 2H, C_6H_4), 7.37 (m, 3H, C_6H_4 + *o*-Se*Ph*), 7.23 (t, *J* = 7.8 Hz, 2H, *m*-Se*Ph*), 6.55 (s, 2H, Mes C*H*), 6.37 (s, 2H, Mes C*H*), 2.12 (s, 3H, Mes *p*-CH_3), 2.10 (s, 3H, Mes *p*-CH_3), 2.01 (s, 6H, Mes *o*-CH_3), 1.78 (s, 6H, Mes *o*-CH_3).

^{13}C NMR (126 MHz, acetone-d_6) δ 165.70 (broad s), 150.72 (broad s), 142.22 (s), 141.41, 140.61 (s), 137.90 (s), 135.24 (s), 133.74 (s), 133.62 (s), 131.95 (s), 131.06 (s), 130.19 (s), 129.98 (s), 129.52 (s), 128.75 (s), 127.83 (s), 126.19 (s), 25.89 (s), 25.32 (s), 20.88 (s), 20.85 (s). Note: one aryl C resonance could not be found, likely due to signal broadening and/or incidental overlap.

^{11}B[^{1}H} NMR (160 MHz, acetone-d_6) δ 14.6 (s).

^{77}Se[^{1}H} NMR (76 MHz, acetone-d_6) δ 943.3 (s).

Elemental analysis calculated for $C_{30}H_{31}BOSe$: C 72.45, H 6.28; found: C 72.29, H 6.25.

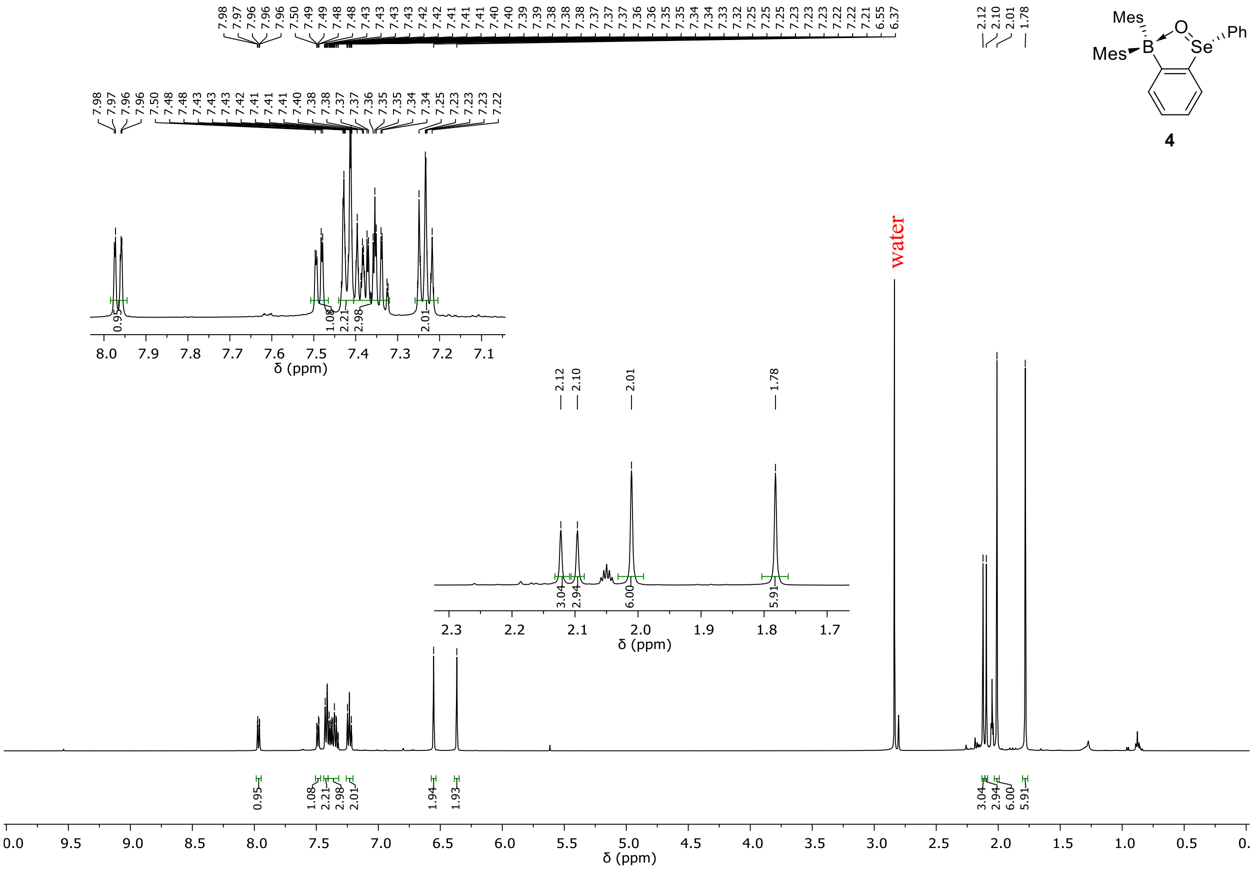

Figure S13. ^{1}H NMR (acetone-d_6, 500 MHz) spectrum of **4**.

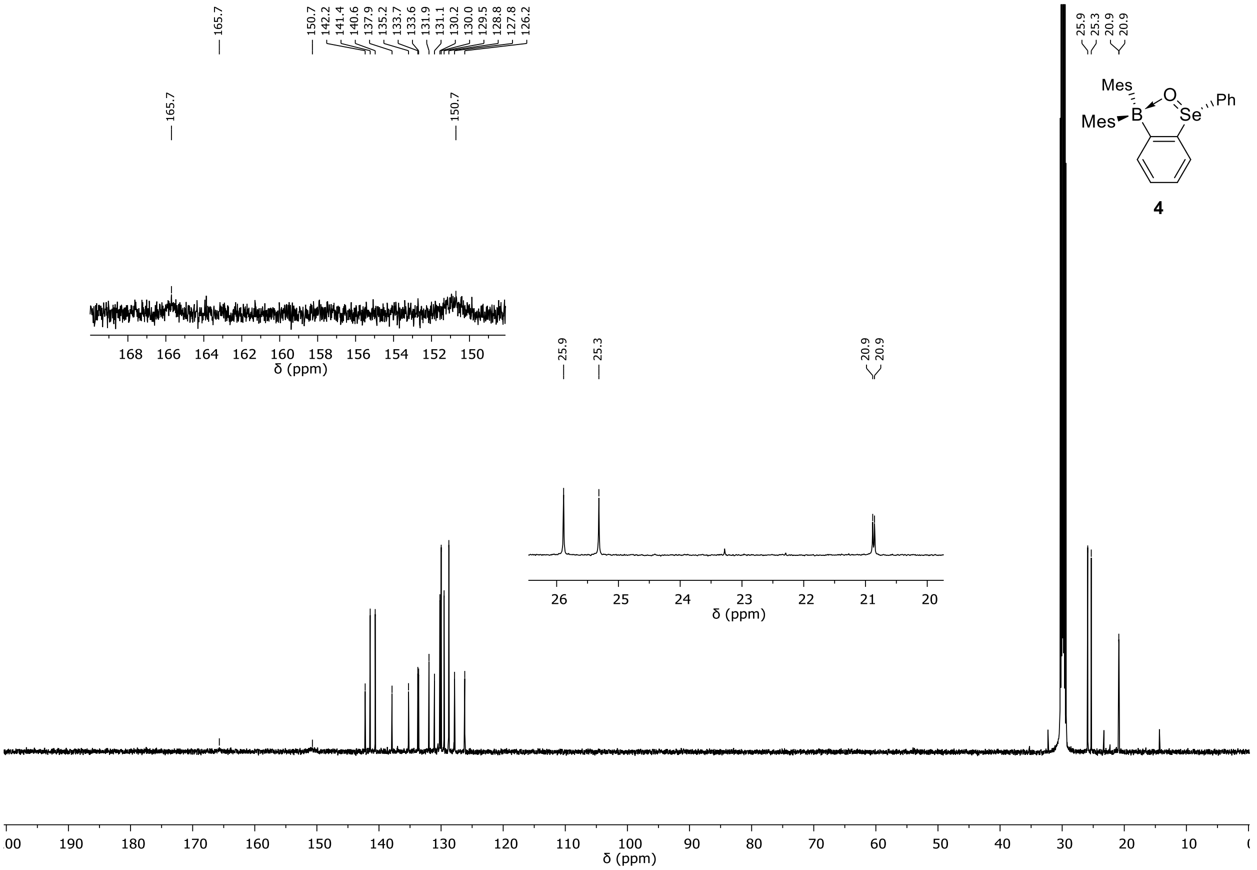

Figure S14. ^{13}C NMR (acetone-d_6, 126 MHz) spectrum of **4**.

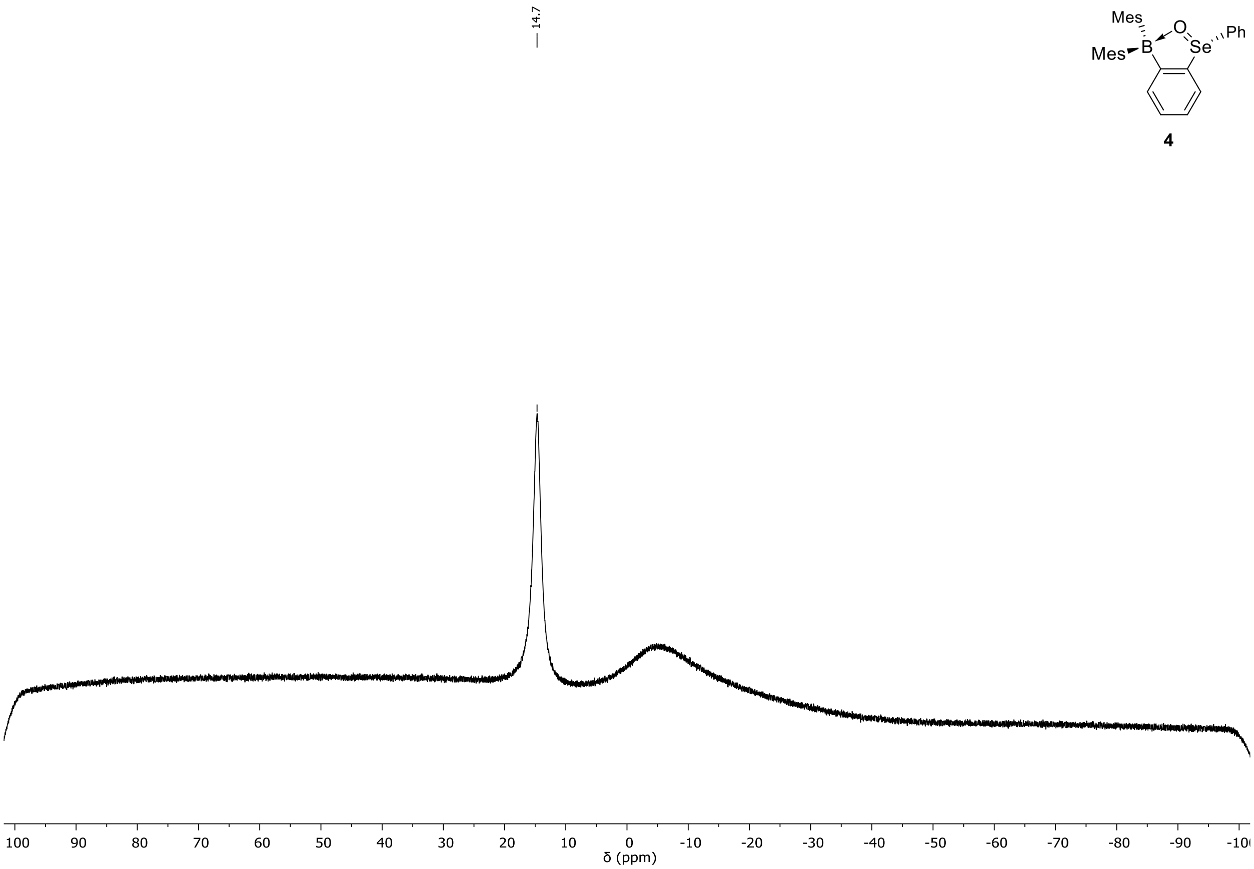

Figure S15. $^{11}B\{^{1}H\}$ NMR (acetone-d_6, 160 MHz) spectrum of **4**.

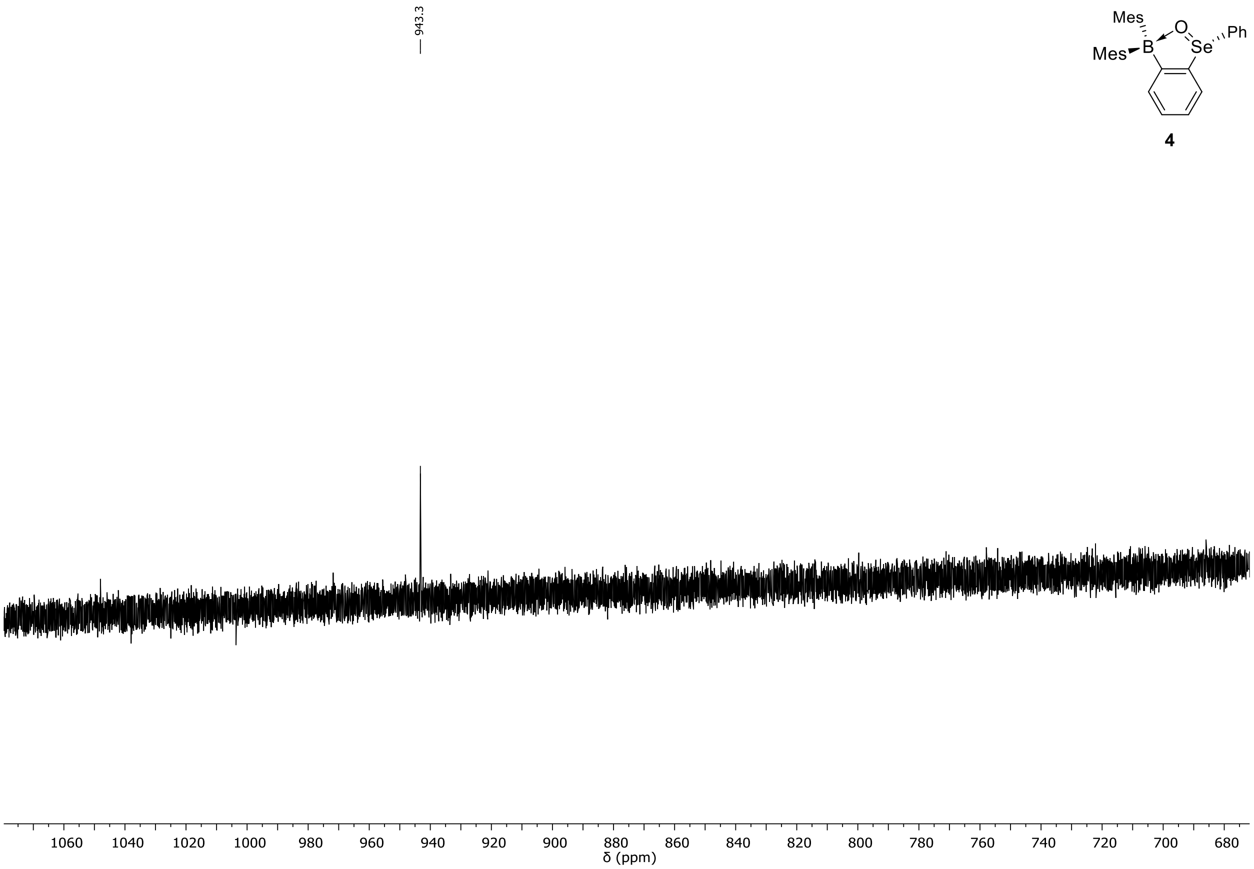

4

Figure S16. $^{77}Se\{^{1}H\}$ NMR (acetone-d_6, 76 MHz) spectrum of **4**.

Synthesis of **5**: In an N_2-filled glovebox, **4** (40.3 mg, 8.1 x 10^{-2} mmol) was dissolved in dry CH_2Cl_2 (5 mL) in a borosilicate glass scintillation vial. Excess HF·pyridine (~1 mL) was added, and the resulting solution stirred overnight. The vial was removed from the glovebox, diluted with water (25 mL), and the aqueous layer was washed with CH_2Cl_2 (3 x 20 mL). The organic layers were combined, dried over $MgSO_4$, filtered, and solvent was removed *in vacuo*. The crude precipitate was then purified *via* vapor diffusion of Et_2O into a $CDCl_3$ solution of the compound, yielding clear, colorless blocks that were collected by filtration. These crystals were suitable for X-ray diffractometry. Yield: 15.4 mg (46%, 3.7 x 10^{-2} mmol).

^{1}H NMR (500 MHz, CD_2Cl_2) δ 7.92 (d, J = 6.7 Hz, 1H, C_6H_4), 7.64 (m, 1H, C_6H_4), 7.56 (m, 3H, C_6H_4 + Se*Ph*), 7.45 (d, J = 7.8 Hz, 2H, *m*-SePh), 7.30 (t, J = 7.8 Hz, 1H, *p*-Se*Ph*), 7.09 (s, 2H, Mes C*H*), 7.07 (d, J = 8.2 Hz, 1H, C_6H_4), 2.37 (s, 3H, Mes *p*- CH_3), 2.21 (s, 6H, Mes *o*-CH_3).

^{13}C NMR (126 MHz, CD_2Cl_2) δ 153.4 (broad s), 144.7 (s), 142.71 (s), 136.2 (d, J = 2.6 Hz) 132.8 (s), 132.7 (s), 132.3 (s), 131.5 (s), 130.7 (s), 129.5 (s), 128.7 (s), 128.4 (s), 127.5 (d, J = 1.6 Hz), 124.0 (d, J = 1.5 Hz), 22.9 (s), 21.4 (s).

^{11}B{^{1}H} NMR (128 MHz, CD_2Cl_2) δ 2.5 (q, $^1J_{B\text{-}F}$ = 47.2 Hz).

^{19}F NMR (470 MHz, CD_2Cl_2) δ -137.9 (broad m).

^{77}Se{^{1}H} NMR (76 MHz, CD_2Cl_2) δ 491 (q, $J_{Se\text{-}F}$ = 50.5 Hz).

HRMS-ESI calculated for $C_{21}H_{20}BF_2Se^+$ $[M\text{-}F]^+$: 401.0786; found: 401.0776.

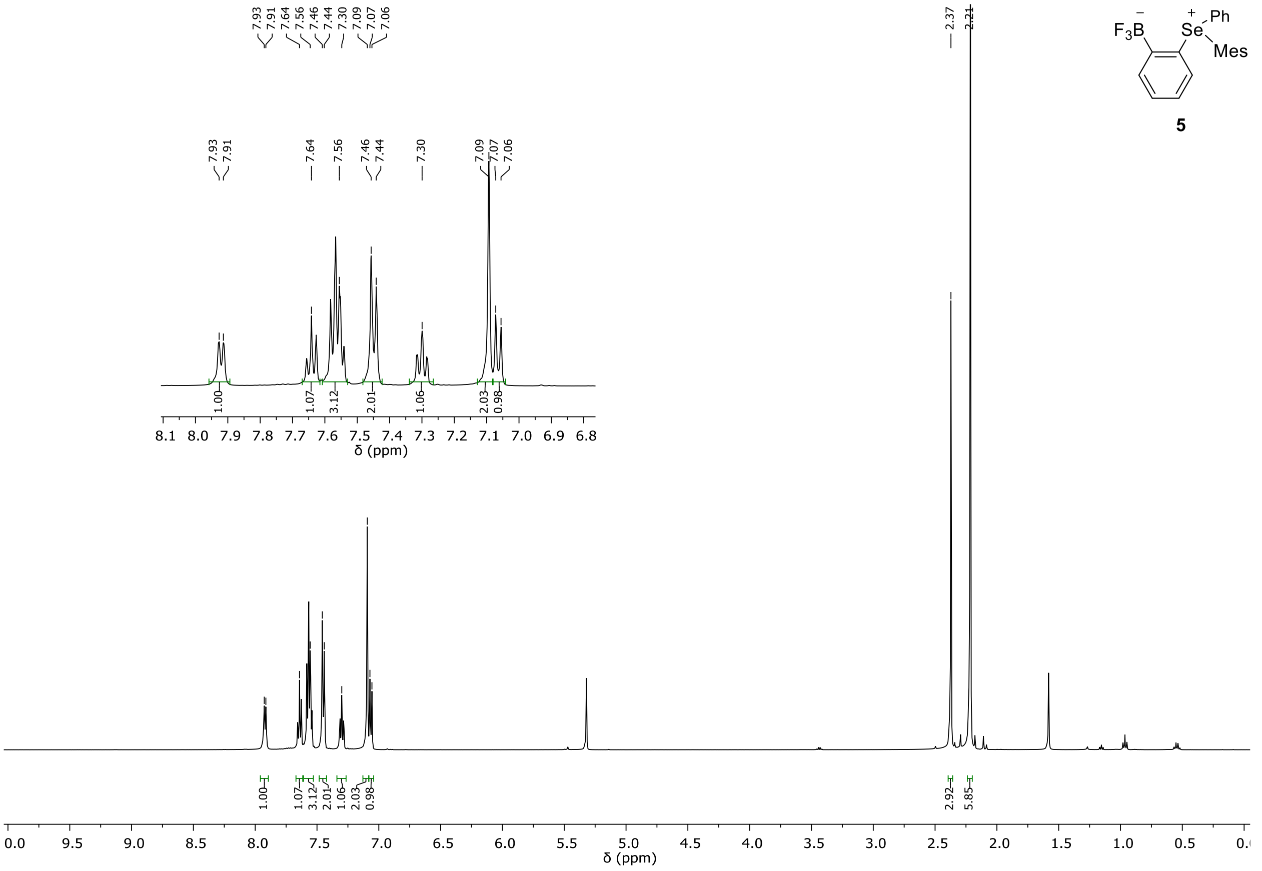

Figure S17. ^{1}H NMR (CD_2Cl_2, 500 MHz) spectrum of **5**.

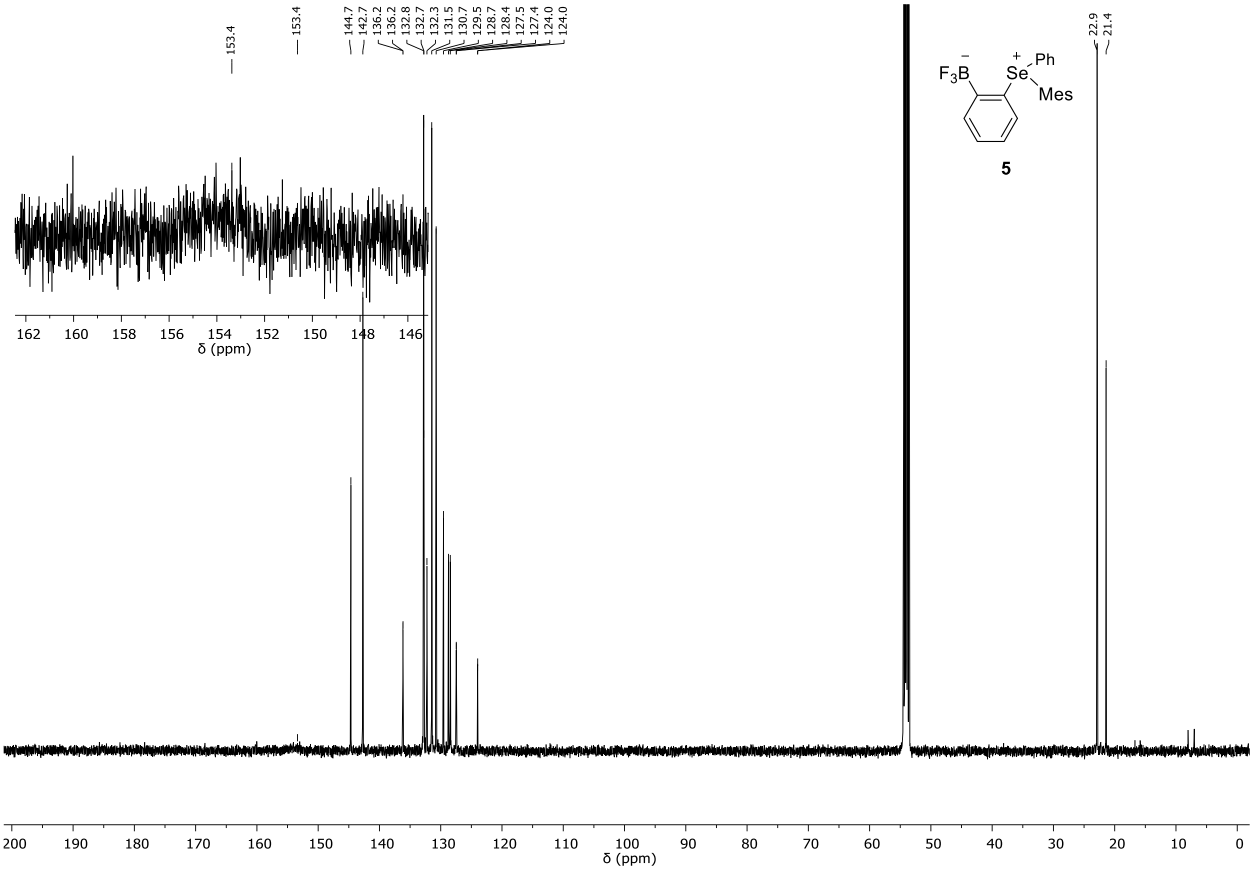

Figure S18. ^{13}C NMR (CD_2Cl_2, 126 MHz) spectrum of **5**. Solvent peak is truncated for clarity.

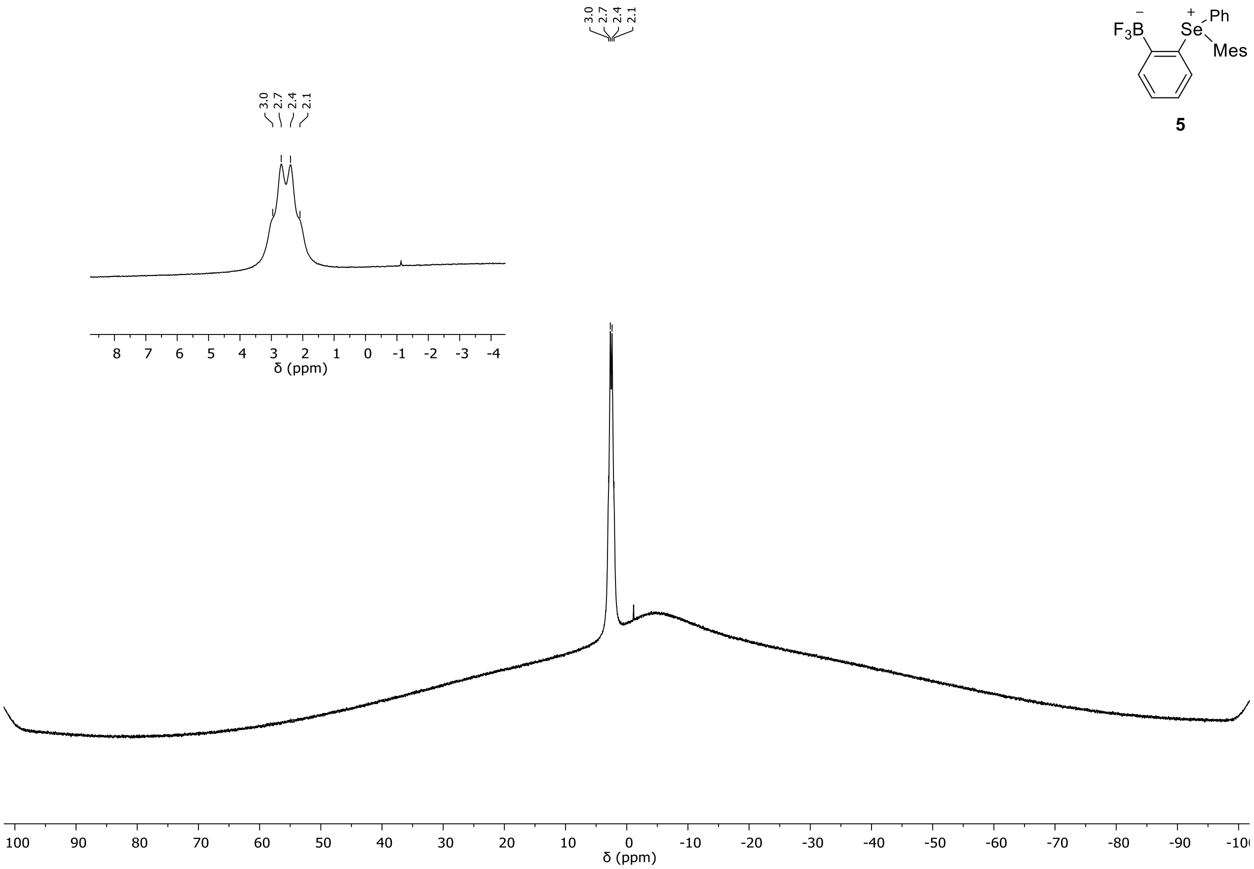

Figure S19. $^{11}B\{^{1}H\}$ NMR (CD_2Cl_2, 160 MHz) spectrum of **5**.

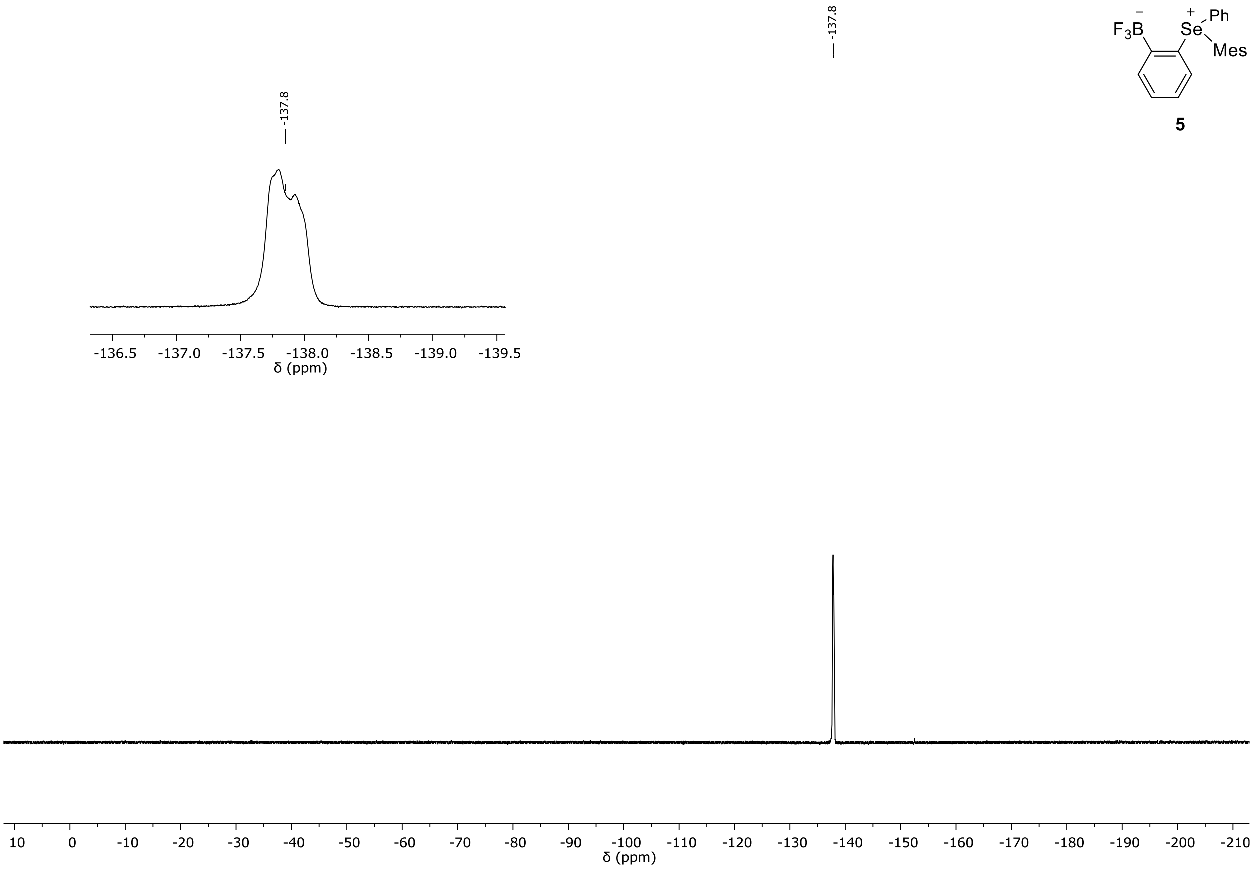

Figure S20. ^{19}F NMR (CD_2Cl_2, 470 MHz) spectrum of **5**.

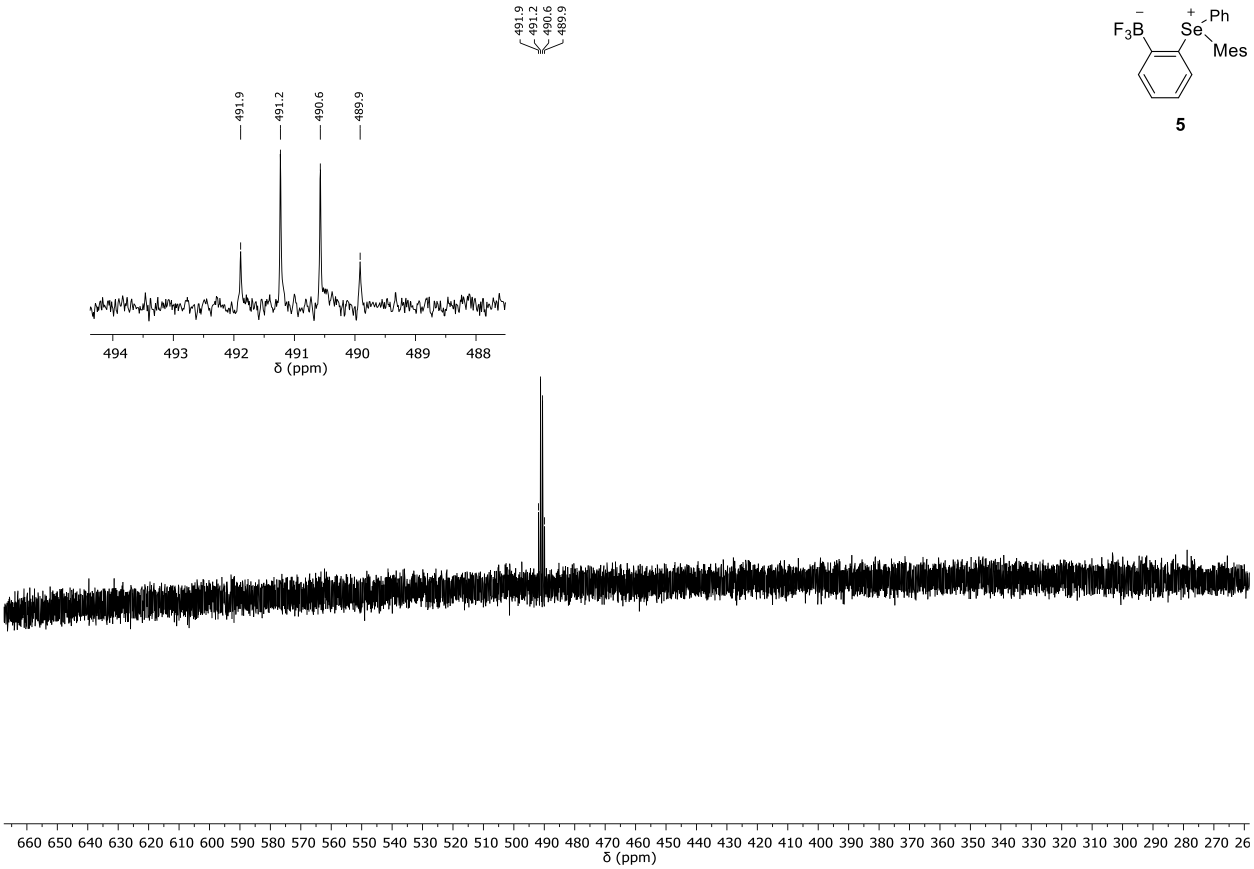

Figure S21. $^{77}Se\{^{1}H\}$ NMR (CD_2Cl_2, 76 MHz) spectrum of **5**.

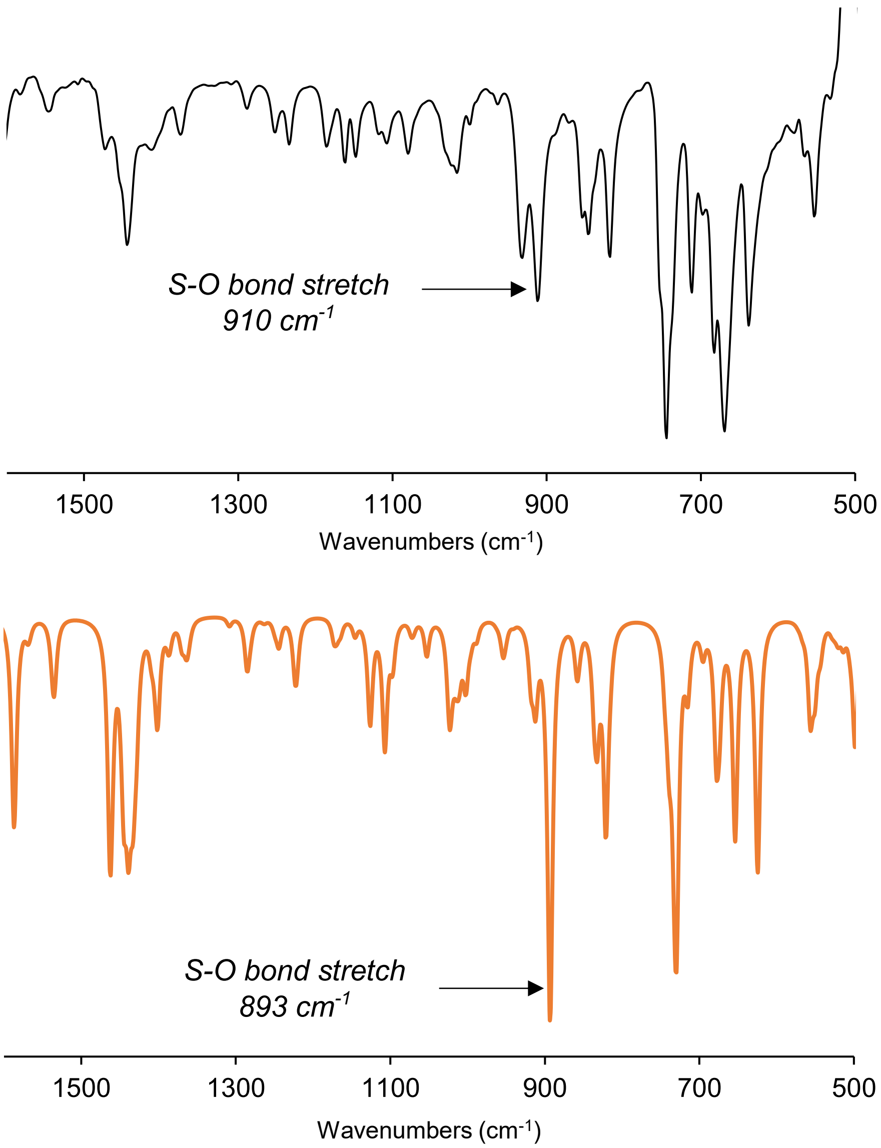

Figure S22. Infrared spectrum of **3** in the solid state (top) and simulated from frequency calculations (bottom). Computational spectrum corrected by multiplication of the harmonic frequencies by 0.963. Details of calculations are found on page S31.

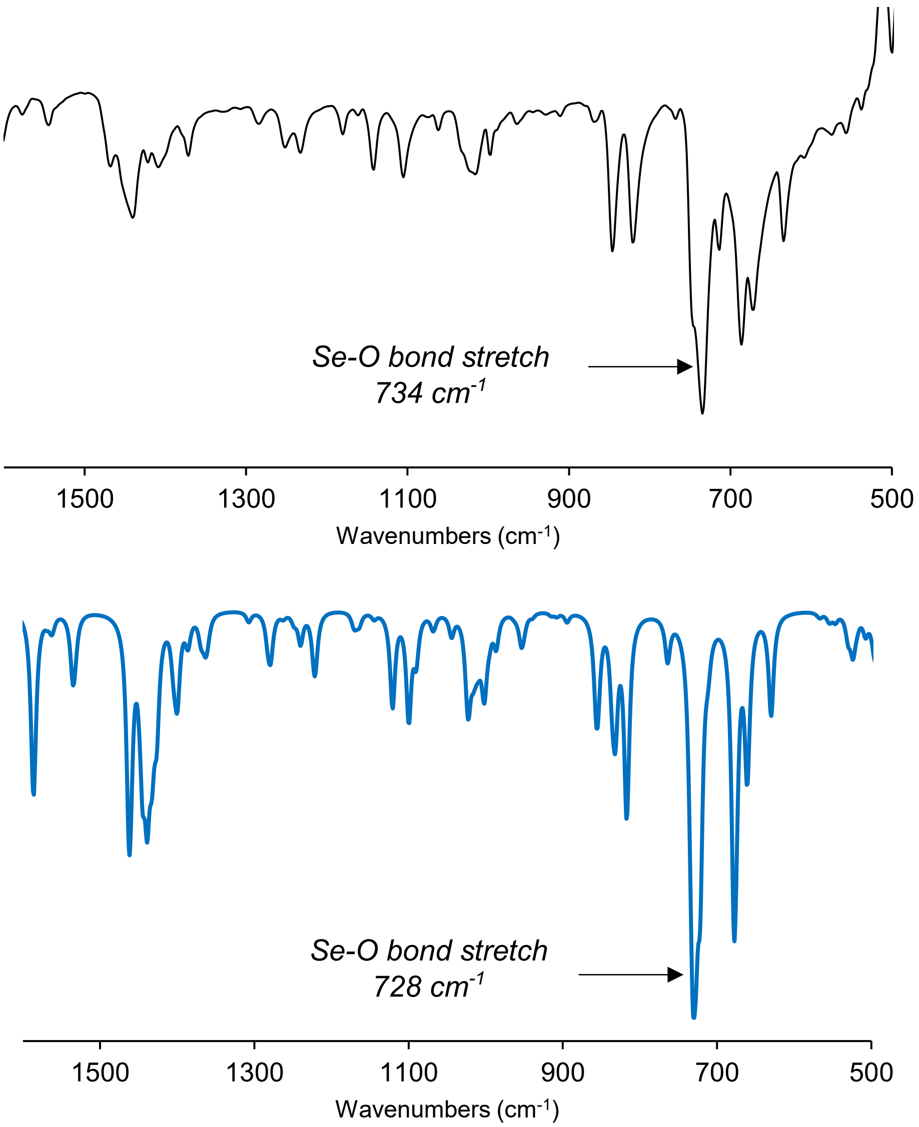

Figure S23. Infrared spectrum of **4** in the solid state (top) and simulated from frequency calculations (bottom), which were corrected by multiplication of the frequencies by 0.963. Details of calculations are found on page S31.

2. Computational details

2.1. General considerations

Density functional theory (DFT) structural optimizations were performed with the Gaussian 16 program.[11] In all cases, the crystal structure geometries were optimized using **Method 1**. Relaxed surface scan calculations were performed using **Method 2** with the optimized geometries described above as starting points. Once the inner and outer forms of **3** were identified, those geometries were again optimized using **Method 1**. Frequency calculations were carried out using that same level of theory to verify the nature of the stationary points for both **3** and **4**.

Using the optimized geometries detailed above, NBO analyses were performed using the Gaussian 16 NBO 7.0 program[12] using **Method 1**. The resulting NBO orbitals were visualized with Avogadro.[13] AIM calculations were performed and visualized using the AIMAll program[14] on the wavefunctions derived from the optimized structures.

Method 1: B3LYP functional[15-16] with GD3BJ empirical dispersion;[17] basis sets: def2tzvp[18] for C/H/O, cc-pVTZ for B/S,[19] and cc-pVTZ-PP for Se.[20]

Method 2: B3LYP functional with application of SMD[21] solvation model of acetonitrile; basis sets: 6-31g(d).

2.2. NBO Output

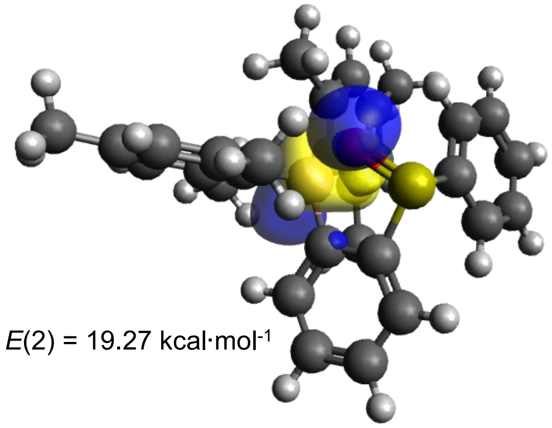

Figure S24. NBOs of the lp(O)→p(B) interactions present in **3** (isovalue 0.05) with associated *E*(2) values.

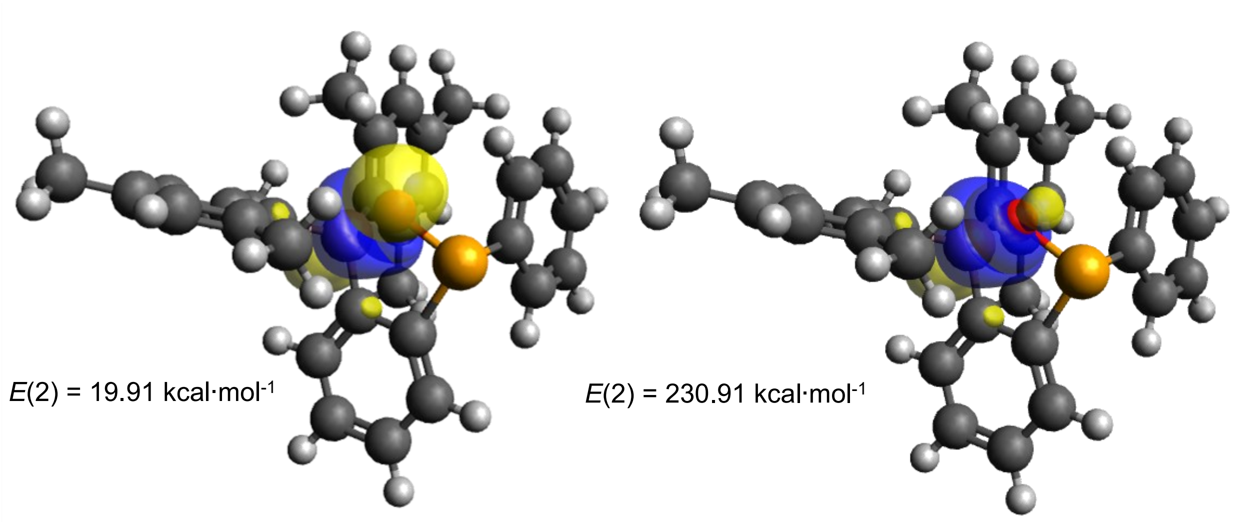

Figure S25. NBOs of the lp(O)→p(B) interaction present in **4** (isovalue 0.05).

2.3. AIM Output

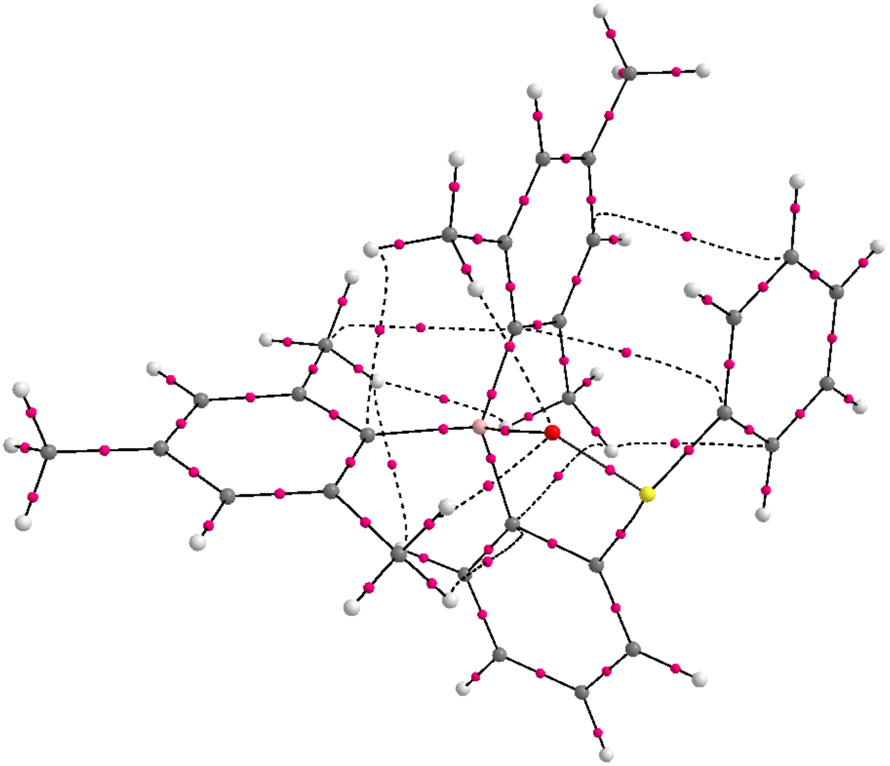

Figure S26. AIM plot showing the bond path between the O and B atoms of **3**.

3. Proposed mechanism for the formation of 5

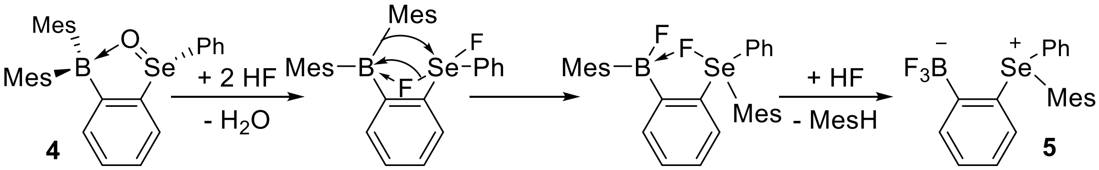

4 **5**

Figure S27. Proposed mechanism for the formation of **5** from **4**.

References

1. Zhao, H.; Kim, Y.; Park, G.; Gabbaï, F. P., Controlling the fluoridophilicity of sulfonium boranes via chelation, Coulombic and hydrophobic effects. *Tetrahedron* **2019**, *75*, 1123-1129.

2. Zhang, J.; Liu, K.; Li, J.; Xie, Y.; Li, Y.; Wang, X.; Xie, X.; Jiao, X.; Tang, B., Harnessing Se=N to develop novel fluorescent probes for visualizing the variation of endogenous hypobromous acid (HOBr) during the administration of an immunotherapeutic agent. *Chem. Commun.* **2021**, *57*, 12679-12682.

3. Rosenau, C. P.; Jelier, B. J.; Gossert, A. D.; Togni, A., Exposing the Origins of Irreproducibility in Fluorine NMR Spectroscopy. *Angew. Chem., Int. Ed.* **2018**, *57*, 9528-9533.

4. Mames, A.; Pietrzak, M.; Bernatowicz, P.; Kubas, A.; Luboradzki, R.; Ratajczyk, T., NMR Crystallography Enhanced by Quantum Chemical Calculations and Liquid State NMR Spectroscopy for the Investigation of Se-NHC Adducts**. *Chem. Eur. J.* **2021**, *27*, 16477-16487.

5. Bruker. *APEX3* v2019.1-0; Bruker AXS Inc.: Madison, WI, 2019.

6. Rigaku Oxford Diffraction, *CrysAlisPro Software System*, version 171.42.61a; Rigaku Corporation: Wroclaw, Poland, 2022.

7. Sheldrick, G. M. *SADABS,* version 2007/4, Bruker Analytical X-ray Systems, Inc.: Madison, WI, 2007.

8. Sheldrick, G. M., SHELXT - Integrated space-group and crystal-structure determination. *Acta Crystallogr., Sect. A: Found. Adv.* **2015,** *71*, 3-8.

9. Sheldrick, G. M. *SHELXL-2014: Program for Crystal Structure Refinement*, University of Göttingen: Germany, 2014.

10. Dolomanov, O. V.; Bourhis, L. J.; Gildea, R. J.; Howard, J. A. K.; Puschmann, H., *OLEX2*: a complete structure solution, refinement and analysis program. *J. Appl. Crystallogr.* **2009,** *42*, 339-341.

11. Frisch, M. J.; Trucks, G. W.; Schlegel, H. B.; Scuseria, G. E.; Robb, M. A.; Cheeseman, J. R.; Scalmani, G.; Barone, V.; Petersson, G. A.; Nakatsuji, H.; Li, X.; Caricato, M.; Marenich, A. V.; Bloino, J.; Janesko, B. G.; Gomperts, R.; Mennucci, B.; Hratchian, H. P.; Ortiz, J. V.; Izmaylov, A. F.; Sonnenberg, J. L.; Williams; Ding, F.; Lipparini, F.; Egidi, F.; Goings, J.; Peng, B.; Petrone, A.; Henderson, T.; Ranasinghe, D.; Zakrzewski, V. G.; Gao, J.; Rega, N.; Zheng, G.; Liang, W.; Hada, M.; Ehara, M.; Toyota, K.; Fukuda, R.; Hasegawa, J.; Ishida, M.; Nakajima, T.; Honda, Y.; Kitao, O.; Nakai, H.; Vreven, T.; Throssell, K.; Montgomery, J. A., Jr.; Peralta, J. E.; Ogliaro, F.; Bearpark, M. J.; Heyd, J. J.; Brothers, E. N.; Kudin, K. N.; Staroverov, V. N.; Keith, T. A.; Kobayashi, R.; Normand, J.; Raghavachari, K.; Rendell, A. P.; Burant, J. C.; Iyengar, S. S.; Tomasi, J.; Cossi, M.; Millam, J. M.; Klene, M.; Adamo, C.; Cammi, R.; Ochterski, J. W.; Martin, R. L.; Morokuma, K.; Farkas, O.; Foresman, J. B.; Fox, D. J. *Gaussian 16*, revision C.01; Gaussian, Inc.: Wallingford, CT, 2016.

12. Glendening, E.D.; Badenhoop, J. K.; Reed, A. E.; Carpenter, J. E.; Bohmann, J. A.; Morales, C. M.; Karafiloglou, P.; Landis, C. R.; Weinhold, F. *NBO 7.0*; Theoretical Chemistry Institute, University of Wisconsin: Madison, WI, 2018.

13. Hanwell, M. D.; Curtis, D. E.; Lonie, D. C.; Vandermeersch, T.; Zurek, E.; Hutchison, G. R., Avogadro: an advanced semantic chemical editor, visualization, and analysis platform. *J. Cheminf.* **2012,** *4*, 17.

14. Keith, T. A. *AIMAll*, version 14.06.21, TK Gristmill Software: Overland Park, KS, 2014.

15. Becke, A. D., Density-functional thermochemistry. III. The role of exact exchange. *J. Chem. Phys.* **1993**, *98*, 5648-5652.

16. Lee, C. T.; Yang, W. T.; Parr, R. G., Development of the Colle-Salvetti Correlation-Energy Formula into a Functional of the Electron-Density. *Phys. Rev. B* **1988**, *37*, 785-789.

17. Grimme, S.; Ehrlich, S.; Goerigk, L., Effect of the damping function in dispersion corrected density functional theory. *J. Comput. Chem.* **2011**, *32*, 1456-1465.

18. Weigend, F.; Ahlrichs, R., Balanced basis sets of split valence, triple zeta valence and quadruple zeta valence quality for H to Rn: Design and assessment of accuracy. *Phys. Chem. Chem. Phys.* **2005,** *7*, 3297-3305.

19. Woon, D. E.; Dunning, T. H., Jr., Gaussian basis sets for use in correlated molecular calculations. III. The atoms aluminum through argon. *J. Chem. Phys.* **1993**, *98*, 1358-1371.

20. Peterson, K. A.; Figgen, D.; Goll, E.; Stoll, H.; Dolg, M., Systematically convergent basis sets with relativistic pseudopotentials. II. Small-core pseudopotentials and correlation consistent basis sets for the post-d group 16-18 elements. *J. Chem. Phys.* **2003**, *119*, 11113-11123.

21. Ribeiro, R. F.; Marenich, A. V.; Cramer, C. J.; Truhlar, D. G., Use of Solution-Phase Vibrational Frequencies in Continuum Models for the Free Energy of Solvation. *J. Phys. Chem. B* **2011,** *115*, 14556-14562.
